# Making Specific Plan Improves Physical Activity and Healthy Eating for Community-Dwelling Patients With Chronic Conditions: A Systematic Review and Meta-Analysis

**DOI:** 10.3389/fpubh.2022.721223

**Published:** 2022-05-19

**Authors:** Hui Lin, Ping Yu, Min Yang, Dan Wu, Zhen Wang, Jiye An, Huilong Duan, Ning Deng

**Affiliations:** ^1^Ministry of Education Key Laboratory of Biomedical Engineering, College of Biomedical Engineering and Instrument Science, Hangzhou, China; ^2^Illawarra Health and Medical Research Institute, University of Wollongong, Wollongong, NSW, Australia; ^3^Department of Nutrition and Food Hygiene, School of Public Health, Chronic Disease Research Institute, Zhejiang University School of Medicine, Hangzhou, China

**Keywords:** physical activity, diet behavior, chronic disease management, implementation intention planning, behavioral interventions

## Abstract

**Background:**

Implementation intention formed by making a specific action plan has been proved effective in improving physical activity (PA) and dietary behavior (DB) for the general, healthy population, but there has been no meta-analysis of their effectiveness for patients with chronic conditions. This research aims to analyze several explanatory factors and overall effect of implementation intention on behavioral and health-related outcomes among community-dwelling patients.

**Methods:**

We searched CIHNAL (EBSCO), PUBMED, Web of Science, Science Direct, SAGE Online, Springer Link, Taylor & Francis, Scopus, Wiley Online Library, CNKI, and five other databases for eligible studies. Random-effects meta-analysis was conducted to estimate effect sizes of implementation intention on outcomes, including PA, DB, weight, and body mass index. And the eligible studies were assessed by the Cochrane Collaboration's tool for risk of bias assessment. Sensitivity analysis adopted sequential algorithm and the p-curve analysis method.

**Results:**

A total of 54 studies were identified. Significant small effect sizes of the intervention were found for PA [standard mean difference (SMD) 0.24, 95% confidence interval (CI) (0.10, 0.39)] and for the DB outcome [SMD −0.25, 95% CI (−0.34, −0.15)]. In moderation analysis, the intervention was more effective in improving PA for men (*p* < 0.001), older adults (*p* = 0.006), and obese/overweight patients with complications (*p* = 0.048) and when the intervention was delivered by a healthcare provider (*p* = 0.01).

**Conclusion:**

Implementation intentions are effective in improving PA and DB for community dwelling patients with chronic conditions. The review provides evidence to support the future application of implementation intention intervention. Besides, the findings from this review offer different directions to enhance the effectiveness of this brief and potential intervention in improving patients' PA and DB.

**Systematic Review Registration:**

https://www.crd.york.ac.uk/PROSPERO/display_record.php?RecordID=160491.

## Introduction

Non-communicable chronic diseases (NCDs) are the leading cause of death, killing 40 million (70%) people globally in 2016. They are the major public health challenge and have caused high economic burden around the world, with an estimated accumulative loss of 30 trillion between 2011 and 2013 according to the World Economic Forum (1). Obesity/overweight [body mass index over 25 kg/m^2^ is considered overweight, and over 30 kg/m^2^ is considered obese (2)] is a well-recognized risk factor that directly impacts mortality and quality of life for people with the above chronic conditions (3).

Two primary risk factors in obesity/overweight are physical inactivity and unhealthy diet, which can cause a range of complications, i.e., heart attack and diabetes (4, 5). The World Health Organization (WHO) defines regular physical activity as moderate intensity physical activity at least 150 min per week for adults (2). Healthy diet involves less sodium intake (2), less fat intake (6, 7), and more intake of fruits and vegetables (8, 9). Regular physical activity and healthy diet can improve blood lipid markers (10, 11), lessen blood pressure (11), and improve psychological wellbeing (12). Thus, they are important facilitators for health and wellbeing of patients with chronic conditions. Improving physical activity (PA) and dietary behavior (DB) is commonly key objectives of behavioral interventions in chronic disease management (13, 14).

Understanding the determinants of behavioral performance is the prerequisite for implementing theory-based behavioral interventions, which subtly influence these determinants and ultimately help people to achieve behavioral goals. “Intention” has long been used by behavioral scientists as a proxy predictor of behavior (15, 16). For example, the theory of planned behavior (TPB) links one's behavioral intention with behavior. It considers behavioral intention as an individual's willingness to act, thus is an immediate predictor of behavior (15). Here, the notion of intention refers to “goal intention” because it connects people's motivations to their behaviors. However, the “intention-action” gap recognizes that strong intentions do not always translate into the corresponding behaviors (17–19). Gollwitzer attributed this gap to ambiguity of goal intention, causing by distraction before initiating the goal behavior and individual forgetfulness. Since goal intention only refers to one's goal behavior and motivations (e.g., “I intend to do more exercise”), its content is often ambiguous and does not attach any situational elements (e.g., when, where, how) to behavior. Every time, individuals who intend to perform the goal behavior have to firstly think when, where or how to act. If the situational content could not be addressed immediately and appropriately, individuals are likely to fall into attention fatigue, and be distracted by another immediately foreseeable rewarding action and give up the original goal (20). This has led to the creation of the concept of implementation intention, which is designed to facilitate the translation of intention into action.

Implementation intention is an explicit form of planning that acts upon elaboration of goal intention *via* specifying the situational content that triggers the goal behavior (21). The mechanism is that, if individuals plan the goal behavior connected with specific situation, then, as long as the situation matches, a person could automatically recollect the planned schema and activate the corresponding behavior. The more concrete the plan is, the less effort is required to activate the needed behavior, which renders the individuals less likely to be distracted (20, 21). The implementation intention intervention is realized by requesting individuals to make concrete behavioral plans by specifying situational elements of “when,” “where,” “how,” e.g., “I plan to do the brisk walking at 3 p.m. at the park near my house 3 times per week,” or making “if-then” statements (22), e.g., “If it is rainy outside, then I will do the brisk walking on the treadmill in the gym nearby.”

Previous meta-analyses studies (19, 23–25), including a large one that analyzed 94 independent studies (25), found that implementation intention has either small to medium or medium to large effects on goal attainment related to healthy eating and exercising among general population. Their pieces of research revealed several factors in intervention design, which could make a difference on the planning effect on PA and DB improvement. For PA, the intervention was favored by combining with barrier management (24) and reinforcement (26), and was more effective in clinical and student samples. While, for DB, the intervention effect was stronger for men than women (19), and in condition when promoting healthy behaviors than diminishing unhealthy ones (23). Other stimulus included was when there was less controlled (23) and no monitoring (19). However, none of the past pieces of research studied the effect on the weight-related outcome and specifically targeted people with chronic conditions. Moreover, there are still underlying moderators to be studied in order to give full play to this intervention.

Due to an acknowledged capacity to fill the “intention-action” gap, and the high demand for effective chronic disease management, there is a high demand for the application of implementation intention intervention to chronic disease management. Compared to the general population, the frequent medical tests and doctor visits, as well as disease symptoms, cause people to have a higher perceived risk (27) and protective motivation (28, 29), and thus a higher intention of health behavior. To date, the published meta-analyses have all been conducted in the general population; further research is required to understand the relevant issues that may impact the effectiveness of implementation intention for patients with chronic disease. For example, what is the influence of the factors, e.g., gender, age, education level, and disease, on the effectiveness of this intervention? What is the best plan pattern, single plan focused on the single behavioral goal, e.g., PA or DB, or multiple plans focused on more than one goal, e.g., both PA and DB? Will the other bundled interventions, i.e., reminders, or different intervention delivery, impact the intervention outcome of patient groups? Will the intervention effect be varied from different follow-up periods? Answers to these questions are important for effective implementation of planning intervention. Therefore, we conducted a systematic review and meta-analysis with a smaller but more focused topic to generate evidence for patients with community-dwelling chronic disease about the effect and potential moderators of implementation intention on improving PA and DB.

## Materials and Methods

The review was conducted according to Cochrane Handbook for Systematic Reviews of Interventions (30) and PRISMA guidelines (31). The checklist was available in Supplementary Table 1. The protocol was registered in the International Prospective Register of Systematic Reviews (PROSPERO: CRD42020160491) prior to undertaking the research. Prior to the registration, we had carried out a feasibility analysis in order to better organize and allocate the research assignment through screening search results against two original eligibility criteria—implementation intention intervention and patients with chronic condition. After consultation and discussion with medical statisticians, we completed the registration, and then the research entered the implementation stage.

### Search Strategies

We searched CIHNAL (EBSCO), PsycInfo (EBSCO), Psychology and Behavioral Sciences Collection (EBSCO), psyARTICLES (EBSCO), MEDLINE (EBSCO), PUBMED, WEB OF SCIENCE, Wiley Online Library, ScienceDirect, SAGE Journals Online, Springer, Taylor & Francis, Scopus for English literature, CNKI, and WANFANG for Chinese literature published during January 1, 1990 to January 1, 2022. The search was focused on identifying RCT that applied implementation intention intervention in chronic disease management. Keywords related to Implementation Intention included “implementation intention,” “action planning,” and “action plan,” and keywords about chronic diseases were modified to suit the different search strategies for databases mentioned above. Details of the search strategies in all databases are presented in Supplementary Table 2. We also searched and identified articles from the reference lists of the included studies and published meta-analyses. Finally, a further search on Google Scholar for the first author of all included studies was conducted to capture any articles that might be missed by the above process.

### Inclusion and Exclusion Criteria

Inclusion criteria were as follows: (1) RCT design; (2) the participants were adult outpatients diagnosed with one or more chronic diseases, including cardiovascular disease, diabetes, chronic lung disease, obese/overweight, and dyslipidemia, etc.; (3) the intervention group received implementation intention interventions aimed at improving PA and/or DB, where the participants were asked to make action plans, detailing the situation and action to achieve the goal. Whereas, there was no restriction on the form (e.g., paper or electronic) or process (with or without the assistance of a healthcare provider) of plan making; (4) outcome measurement included the patients' health behavior or weight outcomes. Studies were excluded when (1) the patients with severe mental disorder, gestation or physical disability to control for factors that are not of research interest; (2) the plan made by the patients failed to meet the principle of implementation intention, or had no specific instruction; (3) no description about the plan form in the original version of the article.

### Data Extraction and Quality Assessment

One reviewer (HL) completed the data extraction and quality assessment of the included studies, and a second reviewer (DW) verified the extracted data. Similarly, disagreements were resolved by consensus with involvement of a third reviewer (ND). Four information items were extracted whenever possible: (1) basic study information, including authors, published year, trial location, and a dependent variable; (2) sample information, i.e., sample size, gender, mean age, education level, and health condition; (3) information about implementation intention intervention, including planned intervention duration, intervention delivery (either delivered by a healthcare provider or fully web based), and a reminder. The latter two were coded as dichotomous data yes/no; and (4) outcome information, including health behavior outcomes (PA and DB), and physiological outcomes (body mass index and weight). The education level was described as the proportion of a well-educated sample (the eighth column), which is assessed in three ways: (1) the proportion of a sample with a high education proportion or education year ≥ 9, (2) education background was General Educational Development (GED) or beyond, (3) not specified but assessed as “high” by the author. The follow-up period was divided by 4 and 28, respectively, when converting the time unit from “day” and “week” into “month.” For studies with multiple measurements of PA or DB, the primary one was extracted and involved into subsequent calculation. If not specified, then the first one being reported in the result part of the original paper was chosen.

Two reviewers (HL and DW) independently assessed the risk of bias in individual studies, applying the Cochrane risk-of-bias tool (30), including: random sequence generation, allocation concealment, blinding of participants and personnel, blinding of outcome assessment, incomplete outcome data, and selective reporting. Each item was rated in three levels: “high risk,” “unclear risk,” or “low risk” in accordance with the instructions in the Cochrane handbook. For each of the six risk items, proportions of studies with low, high, and unclear risk levels were calculated. Only studies with more than three (> 3) low risk items and less than two (<2) high risk items were rated as high-quality studies. Stratified pooled effect sizes were calculated for the high-quality studies. Differences were resolved by consensus among the three reviewers.

### Study Selection

Two reviewers (HL and DW) simultaneously and independently completed the review of titles, abstracts, and full texts after removing duplicates. Handing searching of a reference list and Google Scholar was conducted after first completion of full text identification by the two reviewers independently. Disagreements were resolved through discussion and consensus, together with the third reviewer (ND).

### Meta-Analyses

All meta-analyses were conducted using Stata 12 (Stata Statistical Software, College Station, Texas, United States) (32). Outcome estimates calculation was using random-effect models, respectively, for PA, DB, weight, and body mass index (BMI). According to the guidance from Cochrane Handbook for Systematic Review, it is necessary to standardize the results before comparison of health behavior outcomes (PA and DB) that were measured in a variety of ways in the previous studies. So, we used the standard mean difference (SMD) to represent effect size of health behavior outcomes (i.e., PA and DB) for the included studies, and mean difference (MD) for effect size of an outcome for weight and BMI. By convention, the cutting value of 0.2, 0.5, and 0.8 of SMD suggests “small,” “medium,” or “large” effect size, respectively (33). For studies with repeated measures for each outcome, only the measure with the follow-up period close to the average value was included in the calculation. The average follow-up period was calculated by dividing the sum of the follow-up period of all measurements by the number of measurements. For instance, a total of 17 PA outcome data were extracted from the included studies where the sum of their corresponding follow-up period was 115 months; then, the average value was 6.8 (115/17) months. If a trial measured a patient's PA outcome respectively at 6, 18, and 36 months, only the 6-month outcome would be included in analysis because it was the one most close to the average value of 6.8 months, and *p* < 0.05 was considered statistically significant for all models. And for three-arm RCT studies, if two intervention groups implemented the same planning interventions but were different in other ways, the control group sample was split into two to make up two comparisons. While if two intervention groups implemented different planning interventions, they were combined into one according to the Section 5, Chapter 16, in Cochrane handbook (30).

Heterogeneity among studies for each outcome were assessed by I square, with *p* < 0.01 considered significantly different. I square is measured in percentage, where values of 25, 50, and 75% represent low, moderate, and high heterogeneity (34). Next, a set of single meta regression analyses was performed to the variables that might impact the intervention effect when the number of cases was over 10. The regression analyses were to identify the potential sources of heterogeneity (30). The other purpose was to explore to what extent those variables correlated with the outcome. Egger test was conducted to assess potential bias due to small study effects if cases for each indicator were more than 10, (35) as well as visual inspection of symmetry of funnel plots (36–38).

Sensitivity analyses were undertaken using sequential algorithm and p-curve analysis. The former was done by performing a series of meta-analyses with one study removed each time to assess the reliability of the estimates (39). Besides, we were advised to conduct p-curve analysis, where p-curve refers to the distribution of significant *p*-values (*p* ≤ 0.05) obtained from statistical tests across a group of studies. For the past few years, p-curve analysis has been recommended to test for publication bias (40–43). The bias is closely correlated with the existence of p-hacking, which means that researchers may keep performing statistical analyses on datasets with overlapping observations until they obtain a significant *p*-value from a sequence of *p*-values, and then “selectively” report it (44). This will lead to the p-curve's shape of p-hacked study left skewed, where p-curve's shape of no-effect study is uniform, and of truly effect is right skewed (41, 42, 44). In brief, p-curve provides an intuitive way to estimate the potential risk for publication bias and average power of evidential value of a set of studies. And Simonsohn's team have developed an online app 4.06 and provided a user guide for conveniently conducting the p-curve analysis (www.p-curve.com). However, later researchers found that this method is only robust to the condition where heterogeneity is low, thus is not recommended for the estimates with high heterogeneity (40, 43, 45). So, we made use of the online tool and the guide to perform the analysis merely on the estimates, which was low in heterogeneity [I^2^ <50% accordingly (40)].

## Results

**Figure 1:**
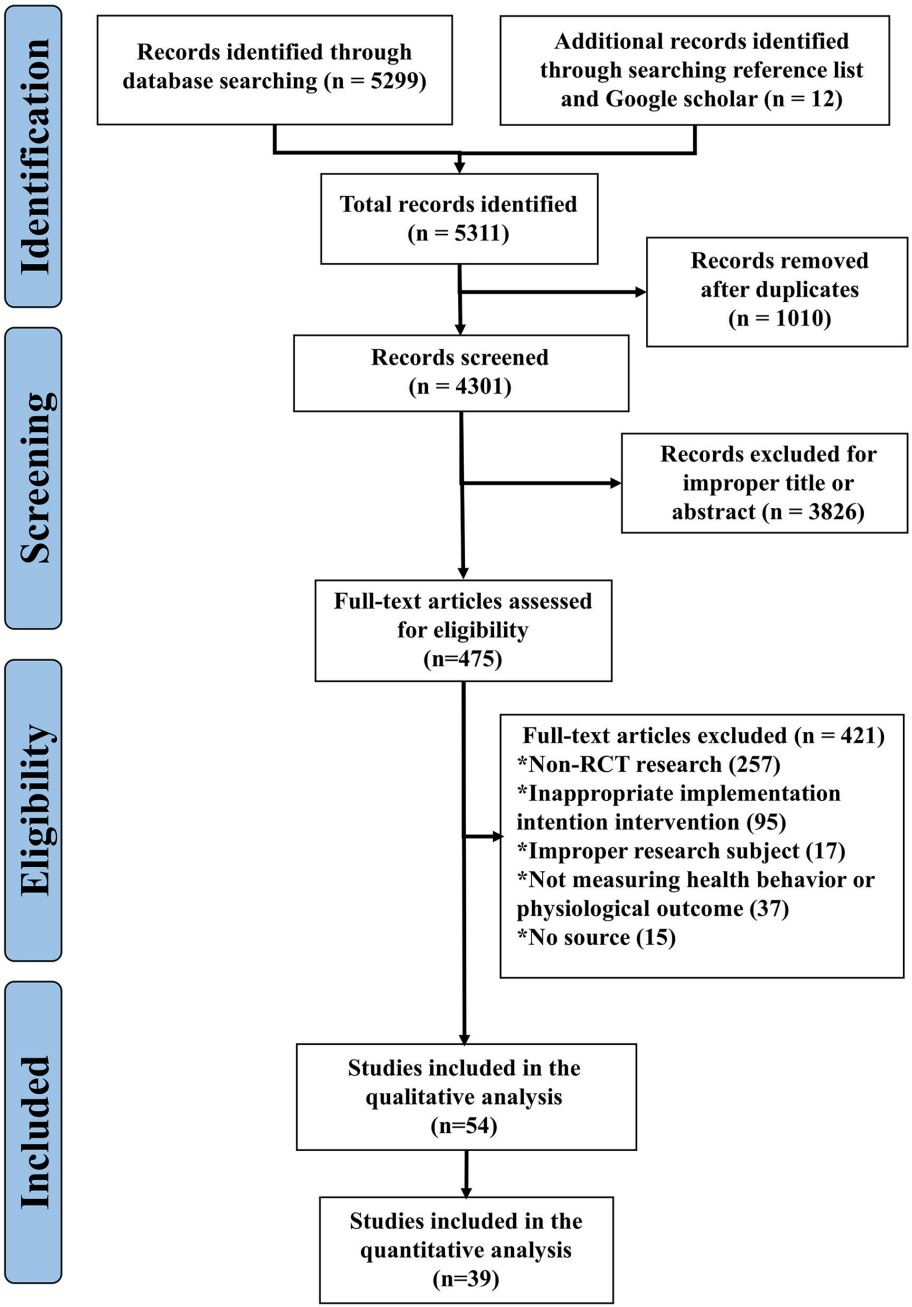
A flow diagram of the article searching process.

A total of 5,299 records published from January 1, 1990 to January 1, 2022 were identified. After removing duplicates, 475 were eligible for full-text review. Additionally, 12 studies were found through further searching the reference lists of the identified articles during data extraction (Figure 1). The full-text screening identified 54 studies that met the inclusion criteria, of which 39 were available for quantitative analysis (Figure 1).

### Study Characteristics

Characteristics of the included studies are summarized in three aspects: basic information, sample characteristics, and interventions (Table 1). Basic information included the first author, country, published year, and dependent variable of the intervention. The majority of studies were conducted in developed countries (73%) and European countries (51%). The country included USA (18%), Netherlands (15%), UK (13%), German (11%), China (11%), Poland (7%), Australia (7%), Brazil (5%), Sweden (5%), Canada (2%), Japan (2%), Kuwait (2%), and Iraq (2%).

**Table 1:** Characteristics of included studies.

**Authors**	**Country**	**DV**	**Sample characteristics**				**Intervention**			
			**Sample size**	**HC**	**Women (%)**	**MA (years)**	**SWE (%)**	**FP (month)**	**Reminder**	**FWD**
Janssen et al. (46)	USA	PA	199	CVD	19	58	26	6, 15	None	No
Bélanger-Gravel et al. (47)	Canada	PA	101	OB	59	59	26	8	None	No
Zakrisson et al. (48)	Sweden	PA	150	COPD, CHF	50	73		3, 12	None	No
Luszczynska (49)	Poland	PA	114	Post-MI	36	54	72	8	None	No
Scholz et al. (50)	German	PA	205	CVD	21	60	67	2.5	None	No
Sniehotta et al. (51)	German	PA	211	CVD	22	59	65	2.5	None	No
Sniehotta et al. (52)	German	PA	240	CVD	19	58	99	2, 4	None	No
Rodrigues et al. (53)	Brazil	PA	136	CVD	36	59		1, 2	Phone call	No
Wilczynska et al. (54)	Australia	PA	84	OB, DM	70	45		2.5, 5	None	Yes
Wooldridge et al. (55)	USA	PA	40	DM	57	55	95	1.5	None	No
Silva et al. (56)	Brazil	PA	65	DM	68	61		3, 6, 12	Hospital visit	No
Engel and Lindner (57)	Australia	PA	54	DM	48	62		3, 6	None	No
Abdolkarimi et al. (58)	Iran	PA	124	DM	78		24	3	Phone call	No
Mayer et al. (59)	USA	PA	402	DM	85	45	55	6	None	No
Peacock et al. (60)	UK	PA	204	OB, HT, DLP, DM	36	64	40	3, 12	None	No
Wurst et al. (61)	German	PA	202	CHD	24	59	32	6, 12	None	No
Kuijer et al. (62)	Netherlands	PA	42	Asthma	69	43		6	None	No
Armitage et al. (63)	Kuwait	DB	216	OB		30		6	None	No
de Freitas Agondi et al. (64)	Brazil	DB	112	HT	100	60		2.5	Phone call	No
Hayes et al. (65)	USA	DB	95	OB	73	21	100	1	Text message	No
Miura et al. (66)	Japan	DB	57	HT	49	62		6	Hospital visit	No
Obara-Golebiowska and Brycz (67)	Poland	DB	100	OB	72			0.5	None	No
Scholz et al. (68)	German	DB	373	OB	72	52	62	6, 12	None	No
Luszczynska, Scholz et al. (69)	Poland	DB	114	Post-MI	36	54	82	8	None	No
Jackson et al. (70)	UK	DB	120	CVD	41	65		0.25, 1, 3.2	None	No
Soureti et al. (71)	UK	DB	781	OB		47		1	None	Yes
Soureti et al. (72)	UK	DB	808	OB		46		1	Text message	Yes
Armitage et al. (73)	UK	DB	72	OB	50	34		1	None	No
Zandstra et al. (74)	Netherlands	DB	57	OB	79	38		1	None	No
Li et al. (75)	China	DB	30	DM	53	41	77	6	None	No
MacPhail et al. (76)	Australia	DB	77	DM		68	98	4	None	No
Liu (77)	China	DB	100	DM	53	55	15	3	None	No
Gao et al. (78)	China	DB	80	DM	49	38	44	6	None	No
Ströbl et al. (79)	German	PA&DB	467	OB, HT, MD, HLP	45	48	34	6, 12	Phone call	No
van Genugten et al. (80)	Netherlands	PA&DB	539	OB	69	48	39	6	Email	Yes
Vinkers et al. (81)	Netherlands	PA&DB	143	OB	41	56	99	12	None	No
Svetkey et al. (82)	USA	PA&DB	1,032	OB, HT, DLP	63	56	62	36	Email, Phone call	Yes
Stevens et al. (83)	USA	PA&DB	1,191	OB	34	43	51	6, 18, 36	None	No
Luszczynska, Sobczyk et al. (84)	Poland	PA&DB	55	OB	100	44	48	2	None	No
Sniehotta et al. (85)	UK	PA&DB	81	OB, HT, etc.	63	57		6	None	No
Thoolen et al. (86)	Netherlands	PA&DB	180	DM	45	62		3, 12	None	No
Duan et al. (87)	China	PA&DB	114	CVD	57	49	89	2	Phone call	Yes
Helena et al. (88)	Sweden	PA&DB	73	OB	21	55	77	6	None	No
Broekhuizen et al. (89)	Netherlands	PA&DB	340	FH	57	45	32	12	Phone call	Yes
Cheung et al. (90)	Netherlands	PA&DB	2,423	OB	58	48	45	6, 12	None	Yes
Nishita et al. (91)	USA	PA&DB	190	DM	63	48		6, 12	None	No
Hardeman et al. (92)	UK	PA&DB	365	DM	62	40		12	None	No
Heredia et al. (93)	USA	PA&DB	168	OB	59	55	55	3, 6	None	No
Heideman et al. (94)	Netherlands	PA&DB	96	DM	68	55	32	3, 9	None	No
Su and Yu (95)	China	PA&DB	146	CHD	16	56		1.5, 3	None	No
Washington et al. (96)	USA	PA&DB	120	DM	67	56	70	6	None	No
Kuijer et al. (62)	Netherlands	PA&DB	62	DM	45	42		6	None	No
Eakin et al. (97)	Australia	PA&DB	434	DM, HT	61	58	45	4, 12	None	No
Jiang et al. (98)	China	PA&DB	500	DM	65	62	5	3, 24	None	No
Swoboda et al. (99)	USA	PA&DB	54	DM	69	56	91	4	None	No

*CHF, congestive heart failure; COPD, chronic obstructive pulmonary disease; CVD, cardiovascular disease; CHD, coronary heart disease; DLP, dyslipidemia; DM, diabetes mellitus; DV, dependent variable; FH, Familial hypercholesterolemia; FP, follow-up period; FWD, fully web-based delivery; HC, health condition; HLP, hyperlipidemia; HT, hypertension; MA, mean age; MD, musculoskeletal disorders; MI, myocardial infarction; SWE, sample of well-educated*.

Sample characteristics were represented by sample size, health condition, gender, mean age, and the educational level. An obese/overweight sample accounted for 30%, obesity with complications for 39%, cardiovascular disease/coronary heart disease/congestive heart failure /hypertension for 33%, diabetes mellitus for 37%, dyslipidemia for the same 9%, chronic lung disease for 4%, and musculoskeletal disorders for 3%. In 29 studies, the female participants outnumbered the male participants. The majority of the subjects in the included studies were middle-aged or older (mean: 52 years; median: 55 years). Of 34 studies which provided education information, 56% had over more than a half well-educated sample.

Information about implementation intention intervention included the follow-up period, reminder form, and delivery form. The follow-up period ranged from 7 days to 36 months. Mean values of the follow-up period for the PA outcome (whose data were accessible) were 6.7 months per measurement. It was 4.7 months per measurement for DB, for BMI was 7.1 months per measurement, and for weight was 11.5 months per measurement. Only 14 studies arranged reminders after planning. Reminder forms included the hospital visit (4%), phone call (13%), text message (4%), and email (4%). Notably, the form of the “hospital visit” refers that the participants received on-site reinforcement of their action plans during the face-to-face follow-up (56, 66). Only 15% of the planning interventions were fully web based; the rest were all delivered by a healthcare provider. Thirty-three studies involved single-plan interventions (17 aimed at improving PA and 16 at improving DB), while the remaining 22 involved multi-plan interventions.

### Quality Assessment

Studies reporting either the PA or DB outcome suffered from a majority of high and unclear risk in blinding of the participants and personnel (84 and 55%, respectively) and selective reporting (58 and 61%, respectively). Half of the studies reporting DB outcome were also at high and unclear risk in blinding of outcome assessment and allocation concealment. A total of nine studies with the PA outcome were assessed as high quality, and the pooled effect size was statistically significant with similarly high heterogeneity [SMD, 0.32; 95% CI (0.09, 0.55), *p* = 0.007, I^2^ = 74%]. Estimated effect size for 10 high-quality studies with the DB outcome was also significant but smaller [SMD −0.18, 95% CI (−0.28, −0.07), *p* < 0.001, I^2^ = 42%]. Pooled effect size for high-quality studies was not statistically significant for either weight or the BMI outcome (both *ps* > 0.05). Quality assessment for the health behavior outcome and risk of bias assessments within individual study is presented in Supplementary Figure 1 and Supplementary Table 3.

### Meta-Analyses

**Figure 2:**
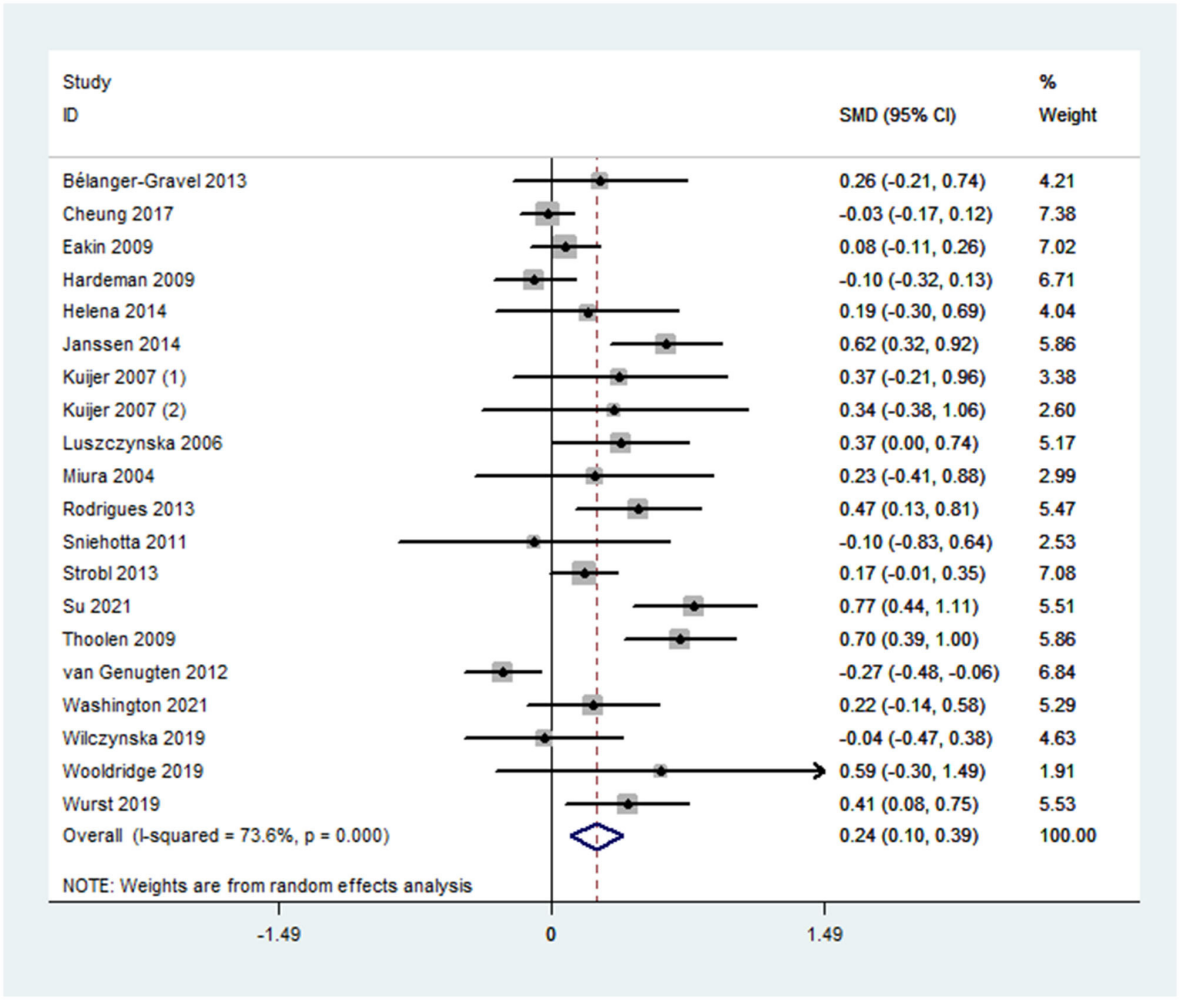
A forest plot of estimated effect sizes for physical activity.

**Figure 3:**
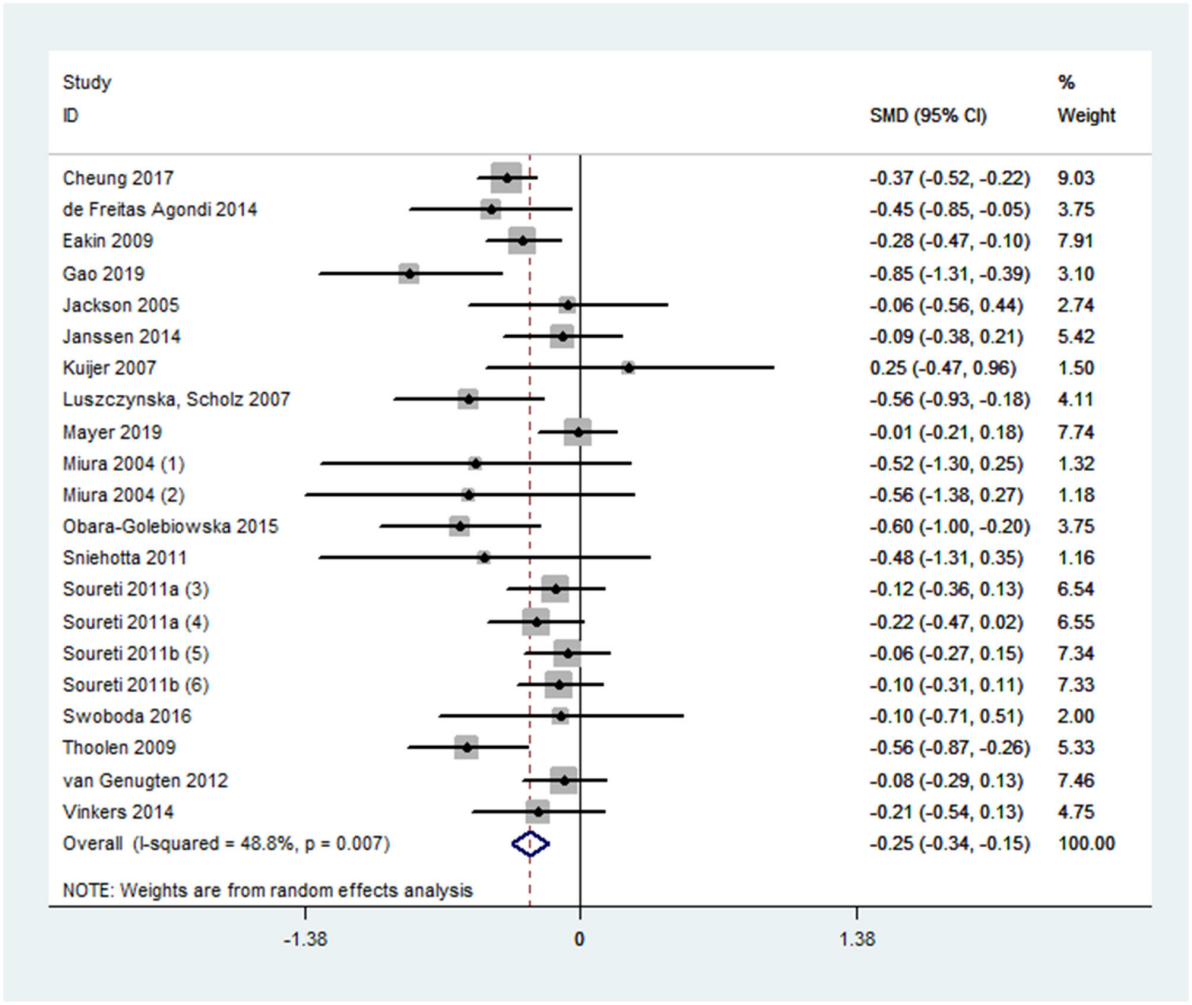
A forest plot of estimated effect sizes for diet behavior.

Overall effect size for PA outcomes calculated from 20 cases was significant yet small [SMD 0.24, 95% CI (0.10, 0.39), *p* < 0.001] (Figure 2). The severity of heterogeneity (*p* < 0.001, I^2^ = 74%) suggested the potential presence of moderators. Overall effect for the DB outcome was significant yet small [SMD, −0.25, 95% CI (−0.31, −0.15], *p* < 0.001]. Random-effect meta-analysis of 21 data sets from 18 studies resulted with a low level of heterogeneity (*p* = 0.007, I^2^ = 49%) (Figure 3). Neither estimates for effect size on BMI (*p* = 0.28) nor weight (*p* = 0.24) were significant. Data information for meta-analyses is available in Supplementary Table 4.

### Moderation Analyses

For the PA outcome, separated meta-regression analysis indicated that the effect of implementation intention was significantly influenced by age, gender, health condition, and intervention delivery (Table 2). Older age [β = 0.03, 95% CI (0.01, 0.05), *p* = 0.006, adjusted R^2^ = 50.48%] was significantly associated with larger effect size. While women [β = −0.01, 95% CI (−0.02, −0.01), *p* = 0.004, Adjusted R^2^ = 83.40%], simple obese or overweight condition [β = −0.33, 95% CI (−0.66, −0.00), *p* = 0.048, Adjusted R^2^ = 26.71%] and fully web-based delivery [β = −0.44, 95% CI (0.76, −0.12), *p* = 0.010, Adjusted R^2^ = 40.13%] had significantly negative correlation with the effect size. No significant moderator was identified either for DB, BMI or weight.

**Table 2:** Moderation analyses for physical activity and diet behavior.

**Moderator variable**	**Estimate**	***SE***	**95% CI**		***p***
			***LL***	***UL***	
**Physical activity**					
Gender (%female)	−0.01	0.00	−0.02	−0.01	**<0.001**
Mean age (year)	0.03	0.01	0.01	0.05	**0.006**
Follow-up period (month)	−0.06	0.03	−0.12	−0.00	**0.045**
Obese/overweight^a^	−0.33	0.16	−0.66	−0.00	**0.048**
Scheduled reminder^b^	−0.16	0.17	−0.53	0.20	0.36
Intervention delivery^c^	−0.44	0.15	−0.76	−0.12	**0.010**
Plan pattern^d^	0.20	0.14	−0.10	0.50	0.18
**Diet behavior**					
Gender (%female)	0.00	0.00	−0.01	0.01	0.83
Mean age (year)	−0.01	0.01	−0.0	0.01	0.25
Follow-up period (month)	−0.01	0.02	−0.04	0.03	0.73
Obese/overweight^a^	0.10	0.10	−0.1	0.31	0.31
Scheduled reminder^b^	0.11	0.11	−0.12	0.34	0.32
Intervention delivery^c^	0.15	0.10	−0.06	0.35	0.14
Plan pattern^d^	0.03	0.11	−0.19	0.25	0.79
**Weight**					
Gender (%female)	0.08	0.04	−0.02	0.17	0.12
Mean age (year)	0.13	0.09	−0.07	0.33	0.19
Follow-up period (month)	0.06	0.07	−0.10	0.21	0.45
Obese/overweight^a^	−3.38	1.57	−6.87	0.11	0.06
Scheduled reminder^b^	2.05	2.17	−2.78	6.87	0.37
Intervention delivery^c^	2.05	2.17	−2.78	6.87	0.37
Plan pattern^d^	0.46	2.19	−4.40	5.32	0.84
**Body mass index**					
Gender (%female)	−0.01	0.01	−0.02	0.02	0.56
Mean age (year)	0.02	0.04	−0.06	0.10	0.55
Follow-up period (month)	−0.10	0.09	−0.29	0.08	0.26
Obese/overweight^a^	0.64	0.34	−0.10	1.38	0.08
Scheduled reminder^b^	0.22	0.39	−0.62	1.07	0.58
Intervention delivery^c^	0.04	0.41	−0.83	0.92	0.92
Plan pattern^d^	0.41	0.49	−0.65	1.46	0.42

*Coding rules:*

a *0 = study sample involved simply obese/overweight people, 1 = study sample did not simply involve obese/overweight people*.

b *0 = no, 1 = yes*.

c *0 = delivered by healthcare provider, 1 = fully web-based delivery*.

d *0 = multiple plan, 1 = single plan*.

*CI, confidence interval; SE, standard error. Bold values indicate significant p values (p < 0.05)*.

### Sensitivity Analyses

Sequential algorithm analyses showed overall modest variations in effect size for PA (between 0.21 and 0.27) and DB (between −0.27 and −0.23), suggesting that the estimates were relatively stable (Supplementary Table 5). The funnel plots were symmetrical for both PA and DB outcomes (Supplementary Figure 2). Results of egger's test were not statistically significant (PA: *p* = 0.12, DB: *p* = 0.26). Since the estimate for PA was accompanied with severe heterogeneity, we had not conducted the p-curve analysis on it. For the DB outcome, descriptively, 88% of all *p-*values were lower than 0.01, and 12% lied in (0.01, 0.02), and the same proportion lied in (0.02, 0.03). None was larger than 0.03. The p-curve was significantly right skewed, indicating evidential value (Supplementary Figure 3). Furthermore, both of the binomial test (*p* = 0.004) and continuous test (full p-curve and half p-curve: *ps* < 0.001) for evidential value [98%, 90% CI (94%, 99%)] were significant. The above findings suggested no significant sign of publication bias.

## Discussion

### Summary of Main Results

To our knowledge, this is the first systematic review and meta-analysis of effects of implementation intention intervention on both PA and DB for community dwelling people with NCD. This review identified 54 studies that applied implementation intention to chronic disease management for community-dwelling outpatients over the world. We studied multiple moderators for the effects of implementation intention intervention for specific groups as recommended (22). Pooled effect sizes for PA and DB were 0.24 [95% CI (0.10, 0.39)] and −0.25 [95% CI (−0.31, −0.15)], respectively, demonstrating significant, small effect of making a specific plan on PA and DB improvement for community-dwelling patients. No significant effect was detected for BMI or weight. Men, older people, and people without obesity/overweight achieved a better PA outcome. Intervention delivered by the healthcare provider has better planning effect than those of fully web based. Delivery by people can enhance the planning effect to improve a DB outcome, whereas a reminder seems to produce negative effect on planning.

### Comparison With Previous Findings

Our result of significant, yet small effect of implementation intention on PA is supported by a previous meta-analysis on both chronic disease and healthy population (24), while in disagreement with another meta-analysis that found no significant results for PA under implementation intention intervention (26). Unlike the moderate effect found among general population (19, 23), there is only small effect on DB improvement among patients with chronic disease. The estimate for a PA outcome was accompanied with high heterogeneity (I^2^ = 76% in this paper), with the previous meta-analyses identifying the existence of a reminder (26), and different planning forms (24), etc., as possible sources of heterogeneity. There can be many factors causing the difference between the population with chronic conditions and normal, healthy population, e.g., gender, age, different initial health conditions and lifestyles. We will further discuss the influences of several factors on intervention effect.

### Interpretation of Findings

It is found that men perform better than women in PA improvement. Different from the finding by Vilà et al. that planning for fat intake reduction is more powerful for men than for women (19), we did not find any gender difference in the DB outcome. Perhaps, it is because our analysis included a broadened scope of DB indicators, which include fat intake (46, 72, 86), fat score, food frequency (66, 85), etc., whereas Vilà et al. only investigated the effect of implementation intention of fat intake behavior (19).

For age, some studies found that weight loss intervention is particularly difficult in young adult population (65, 100). And a high drop-out rate was reported in the younger patient sample in several included studies (74, 79, 80). On the other hand, the reduced effect of implementation intention on PA improvement in the obese/overweight population than the opposite may be explained by a lack of self-awareness of own obese conditions and the associated harm to health (85), the behavioral goal that is not attainable, i.e., desired rapid and remarkable weight loss (81), and a lack of knowledge about strategies to lose weight (65).

As expected, healthcare provider-guided delivery has achieved a better outcome than fully web-based delivery in PA improvement. This finding differed from a previous meta-analysis study that found fully web-based intervention achieved better behavior improvement, such as increased exercise time, increased engagement (101), etc. It is reported that fully web-based intervention tended to have a high rate of “loss to follow-up” or low exposure (72, 81). For example, one study reported that only 15% of the patients finished all of the four intervention modules online (80). Another study found over a 50% missing rate of study population (90). Furthermore, unguided interventions could lead to a lack of goals and poor focused plans (102, 103). Thus, face-to-face support from a healthcare provider for patients has been inferred as a facilitator for effective planning intervention so far.

We did not find any significant effect of a reminder on the implementation intention for PA, opposite to what found in the general population (26). Similarly, there was no effect of a reminder on the DB outcome. Scheduling a plan reminder acts as a prompt for patients to adhere to their plans. There are a variety of forms [e.g., phone calls (64), text messages (71), emails (80)], frequencies, and contents of a reminder. Thus, the homogeneity of the reminder among included studies was far from satisfactory. Furthermore, neither the PA nor the DB outcome is significantly influenced by the plan pattern. Swoboda et al. conducted a study with a single-plan intervention, a multiple-plan intervention, and a blank control group. The descriptive data presented in their study appeared to imply that the single planned intervention model resulted in greater improvement. They did not, however, compare the differences between two plan patterns. Although both exercise and diet improvement are important, it is unclear whether changing multiple behaviors at once or changing different behaviors sequentially is better for patients with chronic disease. Future research should be conducted to compare the outcomes of various plan patterns.

### Limitations

This study has several limitations. We considered only populations with multiple chronic conditions, which limited the generalizability of the findings but allowed us to narrow our focus, whereas, overweight, which has common health consequences to obesity, was also included in this review despite the fact that it is not an illness and does not match the inclusion criteria for patients with chronic disease. Besides, the exclusion of non-RCT studies could have resulted in data loss. However, during the protocol-drafting phase, we believed that including RCTs would provide more unbiased estimates if we could obtain an adequate number of articles. Additionally, because only English or Chinese language studies were included, it was possible for publications in other languages to be overlooked. Furthermore, none of the studies met all Cochrane risk bias quality criteria; quality of evidence was not optimal. Another limitation is that high heterogeneity for physical activity was identified, reflecting the integrity of chronic disease management and high inconsistency in outcome appraisal of implementation intention intervention. For regression analysis, we acknowledged that the regression of gender and age of a sample might have introduced aggregation bias since we did not collect individual patient data. In addition, the study used a novel method, p-curve analysis, as supplementary of sensitivity analysis. We noted that exclusion of non-significant study (*p* > 0.05) was recognized as an inherent limitation of p-curve analysis (39). However, “although excluding non-significant results makes p-curve noisier (that is, less efficient in estimating the real effect size), it does not make p-curve biased” (p. 675). So, in this paper, we only used it as a sensitivity analysis method rather than the method to estimate the overall effect size (37, 40).

## Conclusion

To the best of our knowledge, this is the first meta-analysis on the effect of implementation intention intervention on community dwelling patients with chronic conditions. At a time of growing concern about chronic disease management, our findings support implementation intention as a promising behavior change intervention for both physical activity and dietary behavior improvement, especially for men, older people, and people with chronic disease but without obese/overweight condition in improving physical activity. Support from a healthcare provider was identified as a facilitator for the intervention effect. And no significant influence was found for the follow-up period, plan pattern, or reminder.

However, with the development of internet and communication technology, it is remained to explore finer and more humanized design to realize effective online planning intervention and plan reinforcement, e.g., a human-computer interaction technique, healthcare system involved both people with specific chronic conditions, and a healthcare provider. It is advisable to analyze the influence of different reminders with different forms in terms of frequency, form, and delivery. Furthermore, the quality and the consistency of study design also need to be improved.

## Data Availability Statement

The original contributions presented in the study are included in the article/Supplementary Material, further inquiries can be directed to the corresponding author/s.

## Author Contributions

Database searching was performed by HL and DW. HL, DW, and ND collectively conducted the data analysis and visualization. HL drafted the work, which had been critically revised for important content by PY. All authors contributed to selecting search terms and defining inclusion/exclusion criteria, contributed to the article, and approved the submitted version.

## Funding

This work was supported by the National Key Research and Development Program of China (No. 2020YFC2006405), the Key Research and Development Program of Guangxi Zhuang Autonomous of China (No. 2020AB33002), the Key Research and Development Program of Zhejiang, China (No. 2021C03111), and the Alibaba Cloud.

## Conflict of Interest

The authors declare that the research was conducted in the absence of any commercial or financial relationships that could be construed as a potential conflict of interest.

## Publisher's Note

All claims expressed in this article are solely those of the authors and do not necessarily represent those of their affiliated organizations, or those of the publisher, the editors and the reviewers. Any product that may be evaluated in this article, or claim that may be made by its manufacturer, is not guaranteed or endorsed by the publisher.
