# Supplementary material for: Making Specific Plan Improves Physical Activity and Healthy Eating for Community-Dwelling Patients With Chronic Conditions: A Systematic Review and Meta-Analysis
Source: Front Public Health. 2022 May 19;10:721223. doi: 10.3389/fpubh.2022.721223 (PMC9160833; doi:10.3389/fpubh.2022.721223)
Supplement: Supplementary file 1 [file Table_1.DOC]

Supplemental Table 1. PRISMA Checklist.

| **Section/topic** | **#** | **Checklist item** | **Reported on page #** |
| --- | --- | --- | --- |
| **TITLE** | | |  |
| Title | 1 | **Making specific plan improves physical activity and healthy eating for community-dwelling patients with chronic conditions: a systematic review and meta-analysis** | 1 |
| **ABSTRACT** | | |  |
| Structured summary | 2 | **Abstract**  **Background**. Implementation intention, formed by making specific action plan, has been proven effective in improving physical activity (PA) and dietary behavior (DB) for the general, healthy population, but there has been no meta-analysis of their effectiveness for patients with chronic conditions. This research aims to analyze several explanatory factors and overall effect of implementation intention on behavioral and health-related outcomes among community-dwelling patients.  **Methods**. We searched CIHNAL (EBSCO), PUBMED, Web of Science, ScienceDirect, SAGE Online, SpringerLink, Taylor & Francis, Scopus, Wiley Online Library, CNKI and five other databases for eligible studies. Random-effects meta-analysis was conducted to estimate effect sizes of implementation intention on outcomes including PA, DB, weight and body mass index. And the eligible studies were assessed by the Cochrane Collaboration's tool for risk of bias assessment. Sensitivity analysis adopted sequential algorithm and p-curve analysis method.  **Results**. A total of 54 studies were identified. Significant small effect sizes of the intervention were found for PA (standard mean difference [SMD] 0.24, 95% confidence interval [CI] [0.10, 0.39]) and for DB outcome (SMD -0.25, 95% CI [-0.34, -0.15]). In moderation analysis, the intervention was more effective in improving PA for men (p < 0.001), older adults (p = 0.006) and obese/overweight patients with complications (p = 0.048) and when the intervention was delivered by healthcare provider (p = 0.01).  **Conclusions**. Implementation intentions are effective in improving PA and DB for community dwelling patients with chronic conditions. The review provides evidence to support the future application of implementation intention intervention. Besides, the findings from this review offer different directions to enhance the effectiveness of this brief and potential intervention in improving patients' PA and DB. | 1-2 |
| **INTRODUCTION** | | |  |
| Rationale | 3 | Implementation Intention is an explicit form of planning that acts upon elaboration of goal intention via specifying the situational content that triggers the goal behavior (21). The mechanism is that if individuals plan the goal behavior connected with specific situation, then as long as the situation matches, the person could automatically recollect the planned schema and activate the corresponding behavior. The more concrete the plan, the less effort required to activate the needed behavior, which renders the individuals less likely to be distracted (20, 21). The implementation intention intervention is realized by requesting individuals to make concrete behavioral plans by specifying situational elements of “when”, “where”, “how”, e.g. “I plan to do the brisk walking at 3 pm at the park near my house 3 times per week”, or making “if-then” statements (22), e.g. “If it’s rainy outside, then I will do the brisk walking on the treadmill in the gym nearby”.  Previous meta-analyses studies (19, 23-25), including a large one that analyzed 94 independent studies (25), found that implementation intention has either small to medium or medium to large effects on goal attainment related to healthy eating and exercising among general population. Their researches had revealed several factors in intervention design which could make a difference on the planning effect on PA and DB improvement. For PA, the intervention was favored by combining with barrier management (24) and reinforcement (26), and was more effective in clinical and student samples. While for DB, the intervention effect was stronger for men than women (19), and in condition when promoting healthy behaviors than diminishing unhealthy ones (23). Other stimulus included when there was less controlled (23) and no monitoring (19). However, none of the past researches studied the effect on weight-related outcome and specifically targeted people with chronic conditions. Moreover, there are still underlying moderators to be studied in order to give full play to this intervention. | 3 |
| Objectives | 4 | We conducted a systematic review and meta-analysis with a smaller but more focus topic, to generate evidence for community-dwelling chronic disease patients about the effect and potential moderators of implementation intention on improving PA and DB. | 3 |
| **METHODS** | | |  |
| Protocol and registration | 5 | The protocol was registered in the International Prospective Register of Systematic Reviews (PROSPERO: CRD42020160491) prior to undertaking the research. | 4 |
| Eligibility criteria | 6 | Inclusion criteria were as follows: (1) RCT design; (2) participants were adult outpatients diagnosed with one or more chronic diseases, including cardiovascular disease, diabetes, chronic lung disease, obese/overweight, and dyslipidemia, etc.; (3) the intervention group received implementation intention interventions aimed at improving PA and/or DB, where participants were asked to make action plans detailing the situation and action to achieve the goal. Whereas, there is no restriction on the form (e.g. paper or electronic) or process (with or without the assistance of a healthcare provider) of plan-making; (4) outcome measurement included patients’ health behavior or weight outcomes. | 4 |
| Information sources | 7 | We searched CIHNAL (EBSCO), PsycInfo (EBSCO), Psychology and Behavioral Sciences Collection (EBSCO), psyARTICLES (EBSCO), MEDLINE (EBSCO), PUBMED, WEB OF SCIENCE, Wiley Online Library, ScienceDirect, SAGE Journals Online, Springer, Taylor & Francis, Scopus for English literature, CNKI and WANFANG for Chinese literature published during January 1, 1990 to January 1, 2022. | 4 |
| Search | 8 | Search was focused on identifying RCT that applied implementation intention intervention in chronic disease management. Keywords related to Implementation Intention included "implementation intention", "action planning", "action plan" and keywords about chronic diseases were modified to suit the different search strategies for databases mentioned above. Details of the search strategies in all databases was presented in Supplemental Table S2. | 4 |
| Study selection | 9 | Two reviewers (HL and DW) simultaneously and independently completed the review of titles, abstracts and full texts after removing duplicates. Handing searching of reference list and Google Scholar were conducted after first completion of full text identification by two reviewers independently. Disagreements were resolved through discussion and consensus together with a third reviewer (ND). | 5 |
| Data collection process | 10 | One reviewer (HL) completed the data extraction and quality assessment of the included studies and a second reviewer (DW) verified the extracted data. Similarly, disagreements were resolved by consensus with involvement of a third reviewer (ND). | 4 |
| Data items | 11 | Four information items were extracted whenever possible: (1) basic study information including authors, published year, trial location and dependent variable; (2) sample information, i.e., sample size, gender, mean age, education level and health condition; (3) information about implementation intention intervention, including planned intervention duration, intervention delivery (either delivered by healthcare provider or fully web-based) and reminder. The latter two were coded as dichotomous data yes/no; and (4) outcome information including health behavior outcomes (PA and DB) and physiological outcomes (body mass index and weight). | 5 |
| Risk of bias in individual studies | 12 | Two reviewers (HL & DW) independently assessed the risk of bias in individual studies applying the Cochrane risk-of-bias tool (30) including: random sequence generation, allocation concealment, blinding of participants and personnel, blinding of outcome assessment, incomplete outcome data, and selective reporting. Each item was rated in three levels: "high risk", "unclear risk" or "low risk" in accordance with the instructions in the Cochrane handbook. | 5 |
| Summary measures | 13 | Two reviewers (HL & DW) independently assessed the risk of bias in individual studies applying the Cochrane risk-of-bias tool (30) including: random sequence generation, allocation concealment, blinding of participants and personnel, blinding of outcome assessment, incomplete outcome data, and selective reporting. Each item was rated in three levels: "high risk", "unclear risk" or "low risk" in accordance with the instructions in the Cochrane handbook. | 5 |
| Synthesis of results | 14 | By convention, the cutting value of 0.2, 0.5, and 0.8 of SMD suggests ‘small’, ‘medium’, or ‘large’ effect size, respectively (33). For studies with repeated measures for each outcome, only the measure with the follow-up period close to the average value was included in the calculation. | 5 |

Page 1 of 2

| **Section/topic** | **#** | **Checklist item** | **Reported on page #** |
| --- | --- | --- | --- |
| Risk of bias across studies | 15 | For each of the six risk items, proportions of studies with low, high and unclear risk level were calculated. Only studies with more than three (>3) low risk items and less than two (<2) high risk items were rated as high-quality studies. Stratified pooled effect sizes were calculated for the high-quality studies. Differences were resolved by consensus among three reviewers. | 5 |
| Additional analyses | 16 | Heterogeneity among studies for each outcome was assessed by I square, with p < 0.01 considered significantly different.  Next, a set of single meta regression analyses were performed to the variables that might impact the intervention effect when the number of cases over 10. The regression analyses were to identify the potential sources of heterogeneity (30). The other purpose was to explore to what extent those variables correlated with the outcome. Egger test was conducted to assess potential bias due to small study effects if cases for each indicator were more than 10, (35), as well as visual inspection of symmetry of funnel plots (36-38).  Sensitivity analyses were undertaken using sequential algorithm and p-curve analysis. The former was done by performing a series of meta-analyses with one study removed each time to assess the reliability of the estimates (39). Besides, we were advised to conduct p-curve analysis, where p-curve refers to the distribution of significant p values (p ≤ .05) obtained from statistical tests across a group of studies. | 6 |
| **RESULTS** | | |  |
| Study selection | 17 | A total of 5,299 records published from January 1, 1990 to January 1, 2022 were identified. After removing duplicates, 475 were eligible for full-text review. Additionally, 12 studies were found through further searching the reference lists of the identified articles during data extraction (Figure 1). The full-text screening identified 54 studies that met the inclusion criteria, of which 39 were available for quantitative analysis (Figure 1). | 6 |
| Study characteristics | 18 | Characteristics of the included studies are summarized in three aspects: basic information, sample characteristics and interventions (Table 1). | 7 |
| Risk of bias within studies | 19 | Quality assessment for health behavior outcome and risk of bias assessments within individual study was presented in Supplemental Figure S1 and Table S3. | 7 |
| Results of individual studies | 20 | Data information for meta-analyses were available in Supplemental Table S4. | 7 |
| Synthesis of results | 21 | Overall effect size for PA outcomes calculated from 20 cases was significant yet small (SMD 0.24, 95% CI [0.10, 0.39], p < 0.001) (Figure 2). The severity of heterogeneity (p < 0.001, I2 = 74%) suggested the potential presence of moderators. Overall effect for DB outcome was significant yet small (SMD -0.25, 95% CI [-0.31, -0.15], p < 0.001). Random-effect meta-analysis of 21 data sets from 18 studies resulted with low level of heterogeneity (p = 0.007, I2 = 49%) (Figure 3). Neither estimates for effect size on BMI (p = 0.28) nor weight (p = 0.24) was significant. | 8 |
| Risk of bias across studies | 22 | A total of nine studies with PA outcome were assessed as high quality, and the pooled effect size was statistically significant with similarly high heterogeneity (SMD 0.32, 95%CI [0.09, 0.55], p = 0.007, I2 = 74%). Estimated effect size for ten high-quality studies with DB outcome were also significant but smaller (SMD -0.18, 95% CI [-0.28, -0.07], p < 0.001, I2 = 42%). Pooled effect size for high-quality studies was not statistically significant for either weight or BMI outcome (both ps > 0.05). | 7 |
| Additional analysis | 23 | For PA outcome, separated meta-regression analysis indicated that the effect of implementation intention was significantly influenced by age, gender, health condition and intervention delivery (Table 2). No significant moderator was identified either for DB, BMI or weight.  Sequential algorithm analyses showed overall modest variations in effect size for PA (between 0.21 to 0.27) and DB (between -0.27 to -0.23), suggesting that the estimates were relatively stable (Supplemental Table S5). The funnel plots were symmetrical for both PA and DB outcomes (Supplemental Figure S2). Results of egger’s test were not statistically significant (PA: p = 0.12, DB: p = 0.26). | 8 |
| **DISCUSSION** | | |  |
| Summary of evidence | 24 | This review identified 54 studies that applied implementation intention to chronic disease management for community-dwelling outpatients over the world. We studied multiple moderators for the effects of implementation intention intervention for specific groups as recommended. (22) Pooled effect sizes for PA and DB were 0.24 (95% CI [0.10, 0.39]) and -0.25 (95% CI [-0.31, -0.15]) respectively, demonstrating significant, small effect of making specific plan on PA and DB improvement for community-dwelling patients. No significant effect was detected for BMI or weight. Men, older people and people without obesity/overweight achieved better PA outcome. Intervention delivered by healthcare provider have better planning effect than those of fully web-based. Delivery by people can enhance the planning effect to improve DB outcome. Whereas, reminder seems to produce negative effect on planning. | 8-9 |
| Limitations | 25 | This study has several limitations. we considered only populations with multiple chronic conditions, which limited the generalizability of the findings but allowed us to narrow our focus. Whereas, overweight, which has common health consequences to obesity, was also included in this review despite the fact that it is not an illness and does not match the inclusion criteria for patients with chronic disease. Besides, the exclusion of non-RCT studies could have resulted in data loss. However, during the protocol-drafting phase, we believed that including RCTs would provide more unbiased estimates if we could obtain an adequate number of articles. Additionally, because only English or Chinese language studies were included, it was possible for publications in other languages to be overlooked. Furthermore, none of the studies met all Cochrane risk bias quality criteria, quality of evidence was not optimal. Another limitation is that high heterogeneity for physical activity was identified, reflecting the integrity of chronic disease management and high inconsistency in outcome appraisal of implementation intention intervention. For regression analysis, we acknowledged that the regression of gender and age of sample might have introduced aggregation bias since we did not collect individual patient data. In addition, the study used a novel method, p-curve analysis, as supplementary of sensitivity analysis. We noted that exclusion of nonsignificant study (p > 0.05) was recognized as an inherent limitation of p-curve analysis (39). However, “although excluding nonsignificant results makes p-curve noisier (that is, less efficient in estimating the real effect size), it does not make p-curve biased” (p. 675). So, in this paper, we only used it as a sensitivity analysis method rather than the method to estimate the overall effect size. (37, 40) | 10 |
| Conclusions | 26 | At a time of growing concern about chronic disease management, our findings support implementation intention as a promising behavior change intervention for both physical activity and dietary behavior improvement, especially for men, older people and people with chronic disease but without obese/overweight condition in improving physical activity. Support from healthcare provider was identified as facilitators for the intervention effect. And no significant influence was found for follow-up period, plan pattern or reminder.  However, with the development of internet and communication technology, it is remained to explore finer and more humanized design to realize effective online planning intervention and plan reinforcement, e.g., human-computer interaction technique, healthcare system involved both people with specific chronic conditions and healthcare provider. It is advisable to analyze the influence of different reminders with different forms in terms of frequency, form and delivery. Furthermore, the quality and consistency of study design also needs to be improved. | 10-11 |
| **FUNDING** | | |  |
| Funding | 27 | This work was supported by the National Key Research and Development Program of China (No. 2020YFC2006405), the Key Research and Development Program of Guangxi Zhuang Autonomous of China (No. 2020AB33002), the Key Research and Development Program of Zhejiang, China (No. 2021C03111) and the Alibaba Cloud. | 11 |

*From:*  Moher D, Liberati A, Tetzlaff J, Altman DG, The PRISMA Group (2009). Preferred Reporting Items for Systematic Reviews and Meta-Analyses: The PRISMA Statement. PLoS Med 6(7): e1000097. doi:10.1371/journal.pmed1000097

For more information, visit: **www.prisma-statement.org**.

Page 2 of 2
