# Supplementary material for: Making Specific Plan Improves Physical Activity and Healthy Eating for Community-Dwelling Patients With Chronic Conditions: A Systematic Review and Meta-Analysis
Source: Front Public Health. 2022 May 19;10:721223. doi: 10.3389/fpubh.2022.721223 (PMC9160833; doi:10.3389/fpubh.2022.721223)
Supplement: Supplementary file 2 [file Table_2.DOCX]

Supplementary Material

# Supplemental Table 2. Search strategies for each database.

| **Database** | | **Strategies for combining keywords** |
| --- | --- | --- |
| EBSCO | CIHNAL | "implementation intention" OR "implementation intentions" OR "action planning" OR "action plan" OR "action plans" (all fields)  AND  obese OR overweight OR obesity OR hypertension OR "high blood pressure" OR hypertensive OR "heart disease" OR cardiovascular OR "cardiac rehabilitation" OR atherosclerosis OR "coronary artery disease" OR "myocardial infarction" OR "myocardial ischemia" OR "heart failure" OR stoke OR cerebrovascular OR infarct OR "chronic pulmonary disease" OR copd OR "chronic airway disease" OR coad OR "airflow obstruction" OR "chronic lung disease" OR diabetes mellitus OR diabetic OR niddm OR t2dm OR "chronic disease" OR "chronic condition" (title) |
|  | PsycInfo |  |
|  | Psychology and Behavioral Sciences Collection |  |
|  | psyARTICLES |  |
|  | MEDLINE |  |
| PUBMED | "implementation intention" OR "action planning" OR "if-then" OR "implementation intentions" OR "action plan" OR "action plans" (all fields)  AND  "Cardiovascular Diseases" OR "Diabetes Mellitus, Type 2" OR "Lung Diseases, Obstructive" OR "Chronic Disease" (MeSH Terms) | |
| WEB OF SCIENCE | TI=(obese OR overweight OR obesity OR hypertension OR "high blood pressure" OR hypertensive OR "heart disease" OR cardiovascular OR "cardiac rehabilitation" OR atherosclerosis OR "coronary artery disease" OR "myocardial infarction" OR "myocardial ischemia" OR "heart failure" OR stoke OR cerebrovascular OR infarct OR chronic pulmonary disease OR copd OR chronic airway disease OR coad OR "airflow obstruction" OR chronic lung disease OR diabetes mellitus OR diabetic OR niddm OR t2dm OR "chronic disease" OR "chronic condition")  AND  TS=("implementation intention" OR "action planning" OR "if-then" OR "implementation intentions" OR "action plan" OR "action plans") | |
| Wiley Online Library | "obese OR overweight OR obesity OR hypertension OR "high blood pressure" OR hypertensive OR "heart disease" OR cardiovascular OR "cardiac rehabilitation" OR atherosclerosis OR "coronary artery disease" OR "myocardial infarction" OR "myocardial ischemia" OR "heart failure" OR stoke OR cerebrovascular OR infarct OR "chronic pulmonary disease" OR "chronic airway disease" OR "airflow obstruction" OR "chronic lung disease" OR diabetes mellitus OR diabetic OR "chronic disease" OR "chronic condition"" in Title  AND  ""implementation intention" OR "implementation intentions" OR "action planning" OR "action plan" OR "action plans"" anywhere | |
| ScienceDirect | "implementation intention" OR "implementation intentions" OR "action planning" OR "action plan" OR "action plans" (all fields)  AND  obese OR overweight OR obesity OR hypertension OR "high blood pressure" OR hypertensive OR "heart disease" OR cardiovascular OR "cardiac rehabilitation" OR atherosclerosis OR "coronary artery disease" OR "myocardial infarction" OR "myocardial ischemia" OR "heart failure" OR stoke OR cerebrovascular OR infarct OR "chronic pulmonary disease" OR "chronic airway disease" OR "airflow obstruction" OR "chronic lung disease" OR diabetes mellitus OR diabetic OR "chronic disease" OR "chronic condition" (title) | |
| SAGE Journals Online |  |  |
| Springer |  |  |
| Taylor & Francis |  |  |
| Scopus |  |  |
| CNKI | (主题:执行意图(精确))OR(主题:实施意图(精确))OR(主题:运动计划(精确))OR(主题:饮食计划(精确))  Translation:  (theme: implementation intention) OR (theme: Intention of implementation) OR (theme: exercise) OR (theme: diet) | |
| WANFANG |  |  |
