# Supplementary material for: Making Specific Plan Improves Physical Activity and Healthy Eating for Community-Dwelling Patients With Chronic Conditions: A Systematic Review and Meta-Analysis
Source: Front Public Health. 2022 May 19;10:721223. doi: 10.3389/fpubh.2022.721223 (PMC9160833; doi:10.3389/fpubh.2022.721223)
Supplement: Supplementary file 3 [file Table_3.DOCX]

Supplementary Material

# Supplemental Table 3. Risk of bias assessment results.

| Study | Bias | Reviewers' judgement | Support for judgement |
| --- | --- | --- | --- |
| Broekhuizen et al. 2012 | Random sequence generation (selection bias) | Low risk | Judgment was based on the original text: ...*Participants were randomly assigned to either the control group (n=159) or the intervention group (n=181) through a stratified computerized randomization procedure using Microsoft© Office Access 2003 software…* |
|  | Allocation concealment (selection bias) | Low risk | Judgment was based on the original text: ...*Randomization was concealed…* |
|  | Blinding of participants and personnel (performance bias) | Low risk | Intervention was web-based delivery. |
|  | Blinding of outcome assessment (detection bias) | Unclear risk | No relative description. |
|  | Incomplete outcome data (attrition bias) | Low risk | Judgment was based on the original text: ...*No differences were found between dropouts and participants regarding the baseline characteristics…* Intention-to-treat analysis was conducted. |
|  | Selective reporting (reporting bias) | Low risk | Original data and protocol were provided |
| Bélanger-Gravel et al. 2013 | Random sequence generation (selection bias) | Low risk | Judgment was based on the original text: ...*Computer generated random numbers table generated by the principal investigator before the screening of participants.* |
|  | Allocation concealment (selection bias) | Low risk | Judgment was based on the original text: *...Participants were subsequently screened by the principal investigator, who was blinded to the randomization allocation sequence.* |
|  | Blinding of participants and personnel (performance bias) | High risk | Single-blind. |
|  | Blinding of outcome assessment (detection bias) | Low risk | Outcomes were measured objectively by pedometers. |
|  | Incomplete outcome data (attrition bias) | Low risk | Judgment was based on the original text: ...*The drop-out rate was statistically equivalent in both conditions.* Intention-to-treat analysis was conducted. |
|  | Selective reporting (reporting bias) | Unclear risk | Original data was provided without protocol. |
| Cheung et al. 2017 | Random sequence generation (selection bias) | Low risk | Judgment was based on the original text: ...*Computer-determined sequence.* |
|  | Allocation concealment (selection bias) | Low risk | Judgment was based on the original text: ...*Participants were randomly assigned in a computer-determined sequence to one of the 3 groups online.* |
|  | Blinding of participants and personnel (performance bias) | Low risk | Intervention was web-based delivery. |
|  | Blinding of outcome assessment (detection bias) | Low risk | Outcome assessment was completed by participants themselves online. |
|  | Incomplete outcome data (attrition bias) | High risk | Judgment was based on original text: ...*Compared to highly educated participants, participants with a low educational level were significantly more likely to drop out... With decreasing age, participants were more likely to drop out... In addition, higher attrition was found in participants who had higher levels of goal setting.* |
|  | Selective reporting (reporting bias) | Low risk | Original data and protocol were provided. |
| de Freitas Agondi et al. 2014 | Random sequence generation (selection bias) | Low risk | Judgment was based on the original text: ...*A random-sequence list generated by SAS software.* |
|  | Allocation concealment (selection bias) | Unclear risk | No relative description. |
|  | Blinding of participants and personnel (performance bias) | High risk | Judgment was based on original text: ...*Patients were recruited from different health-care settings, and the intervention was applied by a single nurse, which might have resulted in a bias in the intervention results related to personal delivery.* |
|  | Blinding of outcome assessment (detection bias) | Low risk | Judgment was based on original text: ...*An independent researcher trained in data collection and blinded to the randomization performed data collection at final follow-up measurement.* |
|  | Incomplete outcome data (attrition bias) | Low risk | Judgment was based on the original text: ...*No differences was found between drop-outs and experimental sample for the sociodemographic data and use of antihypertensive medications.* |
|  | Selective reporting (reporting bias) | Unclear risk | Original data was provided without protocol. |
| Duan et al. 2018 | Random sequence generation (selection bias) | Unclear risk | No relative description. |
|  | Allocation concealment (selection bias) | Unclear risk | No relative description. |
|  | Blinding of participants and personnel (performance bias) | Low risk | Intervention was web-based delivery. |
|  | Blinding of outcome assessment (detection bias) | Low risk | Outcome assessment was completed by participants themselves online. |
|  | Incomplete outcome data (attrition bias) | Low risk | Judgment was based on the original text: ...*Participants at T1 and T2 did not significantly differ from dropouts at T2 regarding the gender, relationship status,* |
|  | Selective reporting (reporting bias) | High risk | No original data. |
| Hayes et al. 2020 | Random sequence generation (selection bias) | Low risk | Judgment was based on the original text: ...*A web-based random number generator was used to assign conditions.* |
|  | Allocation concealment (selection bias) | Unclear risk | No relative description. |
|  | Blinding of participants and personnel (performance bias) | Low risk | Judgment was based on original text: ...*Both the experimenter and the participant were blind to the assignment prior to this point.* |
|  | Blinding of outcome assessment (detection bias) | Unclear risk | No relative description. |
|  | Incomplete outcome data (attrition bias) | High risk | Judgment was based on the original text: ...*For missing data analysis, no baseline variables were related to missing posttreatment variables, with two exceptions…* |
|  | Selective reporting (reporting bias) | High risk | No original data (data was not separated by groups). |
| Helena et al. 2014 | Random sequence generation (selection bias) | Low risk | Judgment was based on the original text: ...*The randomization procedure was made in blocks of 10 using a random integer generator (www.random.org).* |
|  | Allocation concealment (selection bias) | Low risk | Judgment was based on the original text: ...*Participant was randomly allocated to one of the two study conditions by a senior researcher not involved in the assessment procedures.* |
|  | Blinding of participants and personnel (performance bias) | Unclear risk | No relative description. |
|  | Blinding of outcome assessment (detection bias) | Low risk | Judgment was based on original text: ...*The nurse responsible for measurements was blinded to the study conditions on both occasions.* |
|  | Incomplete outcome data (attrition bias) | Low risk | Intention-to-treat analysis was conducted. |
|  | Selective reporting (reporting bias) | Unclear risk | Original data was provided without protocol. |
| Jackson et al. 2005 | Random sequence generation (selection bias) | Unclear risk | No relative description. |
|  | Allocation concealment (selection bias) | Low risk | Judgment was based on the original text: ...*The allocation was by sealed envelope procedure in which the recruiting researcher could not predict the participant's group.* |
|  | Blinding of participants and personnel (performance bias) | Unclear risk | Judgment was based on original text: ...*Participants were unaware of the alternative interventions.* Whether it was double-blind trial could not be confirmed |
|  | Blinding of outcome assessment (detection bias) | Unclear risk | No relative description. |
|  | Incomplete outcome data (attrition bias) | Low risk | Judgment was based on the original text: ...*There were no significant differences between the participants who completed the study and those who did not in terms of gender, age, daily fruit and vegetable consumption or intention at recruitment.* |
|  | Selective reporting (reporting bias) | Unclear risk | Original data was provided without protocol. |
| Janssen et al. 2014 | Random sequence generation (selection bias) | Low risk | Blocked randomization was conducted. |
|  | Allocation concealment (selection bias) | Low risk | Judgment was based on the original text: ...*Randomization was carried out by the coordinating secretariat using opaque sealed envelopes.* |
|  | Blinding of participants and personnel (performance bias) | Low risk | Judgment was based on original text: ...*Identical questionnaires and structured interview formats were used for both groups, so that the health psychologists could be kept blind to treatment allocation.* |
|  | Blinding of outcome assessment (detection bias) | Low risk | Judgment was based on original text: ...*Follow-up assessments were carried out by trained health psychologists who were blind to group assignment* |
|  | Incomplete outcome data (attrition bias) | Low risk | Judgment was based on the original text: ...*A series of t-tests and Pearson's Chi squared tests showed that the non-participants did not differ significantly from the participants on demographic characteristics...* Intention-to-treat analysis was conducted. |
|  | Selective reporting (reporting bias) | Unclear risk | Original data was provided. |
| Luszczynska et al. 2006 | Random sequence generation (selection bias) | Low risk | Randomization check was conducted. |
|  | Allocation concealment (selection bias) | Unclear risk | No relative description. |
|  | Blinding of participants and personnel (performance bias) | Unclear risk | No relative description. |
|  | Blinding of outcome assessment (detection bias) | Unclear risk | No relative description. |
|  | Incomplete outcome data (attrition bias) | Unclear risk | No analysis was conducted for dropouts. |
|  | Selective reporting (reporting bias) | Unclear risk | Original data was provided without protocol. |
| Luszczynska, Scholz et al. 2007 | Random sequence generation (selection bias) | Low risk | Random number sequence (generated by a random digit generator) was used. |
|  | Allocation concealment (selection bias) | Low risk | Judgment was based on the original text: ...*The experimenters who assigned participants to the groups were not the same as those who created the number sequence or those who delivered the intervention.* |
|  | Blinding of participants and personnel (performance bias) | Low risk | Judgment was based on original text: ...*Participants were not aware of their group assignment. The experimenters who delivered intervention-group and control-group procedures were blinded to group assignment.* |
|  | Blinding of outcome assessment (detection bias) | High risk | Judgment was based on original text: *Outcome assessors were not blinded, which could have an effect on responses collected at T3.* |
|  | Incomplete outcome data (attrition bias) | Low risk | Judgment was based on the original text: ...*Dropout was not dependent on the core variables of the study…* |
|  | Selective reporting (reporting bias) | Unclear risk | Original data was provided without protocol. |
| Luszczynska, Sobczyk et al. 2007 | Random sequence generation (selection bias) | Low risk | Random number sequence (generated by a random digit generator) was used. |
|  | Allocation concealment (selection bias) | Low risk | Judgment was based on the original text: ...*After completing preintervention questionnaires, which included personalized codes to allow matching but maintain anonymity…* |
|  | Blinding of participants and personnel (performance bias) | Unclear risk | Judgment was based on original text: ...*Participants were not aware of their group assignment, and the researchers who delivered the intervention were not those who measured weight and height.* Whether it was double-blind trial could not be confirmed |
|  | Blinding of outcome assessment (detection bias) | Low risk | Judgment was based on original text: ...*Experimenters assessing body weight and height were blinded to group allocation.* |
|  | Incomplete outcome data (attrition bias) | Low risk | Judgment was based on the original text: ...*Data were analyzed on a complete case basis, with pairwise deletion of missing cases.* |
|  | Selective reporting (reporting bias) | Unclear risk | Original data was provided without protocol. |
| Miura et al. 2004 | Random sequence generation (selection bias) | High risk | Differences were observed in the baseline characteristics of the three groups. |
|  | Allocation concealment (selection bias) | Unclear risk | No relative description. |
|  | Blinding of participants and personnel (performance bias) | Unclear risk | No relative description. |
|  | Blinding of outcome assessment (detection bias) | Unclear risk | No relative description. |
|  | Incomplete outcome data (attrition bias) | Low risk | Only one dropout. |
|  | Selective reporting (reporting bias) | High risk | No original data. |
| Obara-Golebiowska et al. 2015 | Random sequence generation (selection bias) | Unclear risk | No relative description. |
|  | Allocation concealment (selection bias) | Unclear risk | No relative description. |
|  | Blinding of participants and personnel (performance bias) | Unclear risk | No relative description. |
|  | Blinding of outcome assessment (detection bias) | Unclear risk | No relative description. |
|  | Incomplete outcome data (attrition bias) | Low risk | No dropout. |
|  | Selective reporting (reporting bias) | High risk | No original data. |
| Rodrigues et al. 2013 | Random sequence generation (selection bias) | Low risk | Judgment was based on the original text: ...*The patients randomized in CG and IG did not differ at baseline.* |
|  | Allocation concealment (selection bias) | Unclear risk | No relative description. |
|  | Blinding of participants and personnel (performance bias) | Unclear risk | No relative description. |
|  | Blinding of outcome assessment (detection bias) | Unclear risk | No relative description. |
|  | Incomplete outcome data (attrition bias) | Low risk | Judgment was based on original text: ...*The 8 patients who dropped out did not differ from the others …* |
|  | Selective reporting (reporting bias) | Unclear risk | Original data was provided without protocol. |
| Scholz et al. 2007 | Random sequence generation (selection bias) | Low risk | Judgment was based on the original text: ...*No baseline differences were found for age, sex, socio-demographics, behavioral intentions, action planning, coping planning, or physical activity among 3 groups.* |
|  | Allocation concealment (selection bias) | Low risk | Judgment was based on the original text: ...*Each participant gave informed consent and received a personal code to match the data of the questionnaires to ensure anonymity.* |
|  | Blinding of participants and personnel (performance bias) | Unclear risk | No relative description. |
|  | Blinding of outcome assessment (detection bias) | Low risk | Judgment was based on original text: ...*For the second assessment, 2 months after discharge, the questionnaires were mailed to the participants at home.* |
|  | Incomplete outcome data (attrition bias) | Low risk | Judgment was based on the original text: ...*Comparing participants who did not complete T2 with those who did yielded no significant differences in age…* |
|  | Selective reporting (reporting bias) | High risk | No original data. |
| Scholz et al. 2013 | Random sequence generation (selection bias) | Low risk | Randomization was unrestricted. But randomization check was conducted. |
|  | Allocation concealment (selection bias) | Low risk | Judgment was based on the original text: ...*Baseline assessment took place in the lab and included informed consent and providing respondents with a personal code to match the data of the questionnaires and 24 h recalls in order to ensure anonymity.* |
|  | Blinding of participants and personnel (performance bias) | High risk | Single-blind. |
|  | Blinding of outcome assessment (detection bias) | Unclear risk | No relative description. |
|  | Incomplete outcome data (attrition bias) | High risk | Judgment was based on the original text: ...*Dropouts reported lower action control than continuers...Dropouts were younger, and had a higher BMI at baseline than continuers... Married individuals were less likely and divorced individuals more likely to drop out.* |
|  | Selective reporting (reporting bias) | High risk | No original data. |
| Sniehotta et al. 2005 | Random sequence generation (selection bias) | Low risk | Randomization check was conducted. |
|  | Allocation concealment (selection bias) | Low risk | Judgment was based on the original text: ...*Every participant was given a personal code to match the data of the questionnaires of the 3 waves to ensure anonymity.* |
|  | Blinding of participants and personnel (performance bias) | Unclear risk | No relative description. |
|  | Blinding of outcome assessment (detection bias) | Unclear risk | No relative description. |
|  | Incomplete outcome data (attrition bias) | Unclear risk | No analysis was conducted for dropouts. |
|  | Selective reporting (reporting bias) | High risk | No original data. |
| Sniehotta et al. 2006 | Random sequence generation (selection bias) | Low risk | Randomization check was conducted. |
|  | Allocation concealment (selection bias) | Unclear risk | No relative description. |
|  | Blinding of participants and personnel (performance bias) | Unclear risk | No relative description. |
|  | Blinding of outcome assessment (detection bias) | Unclear risk | No relative description. |
|  | Incomplete outcome data (attrition bias) | Low risk | Judgment was based on the original text: ... *The 35 patients who dropped out did not differ from the others.* |
|  | Selective reporting (reporting bias) | Unclear risk | Original data was provided without protocol. |
| Sniehotta et al. 2011 | Random sequence generation (selection bias) | Low risk | Judgment was based on the original text: ...*Individual computer-generated randomization to intervention or control condition in a 2:1 ratio, using a secure centralized web-based randomization system provided by the Health Services Research Unit (HSRU)…* |
|  | Allocation concealment (selection bias) | High risk | Judgment was based on the original text: ...*Participants and intervention facilitator were aware of condition allocations... Returning participants at follow-up assessment were asked not to disclose allocation* |
|  | Blinding of participants and personnel (performance bias) | High risk | Judgment was based on original text: ...*Participants and intervention facilitator were aware of condition allocations.* |
|  | Blinding of outcome assessment (detection bias) | Low risk | Judgment was based on original text: ...*The outcome assessor was blinded to allocation.* |
|  | Incomplete outcome data (attrition bias) | Low risk | Intention-to-treat analysis was conducted. |
|  | Selective reporting (reporting bias) | Low risk | Original data and protocol were provided. |
| Soureti et al. 2011a | Random sequence generation (selection bias) | Low risk | Judgment was based on the original text: ...*participants who returned to the website were randomly allocated, using a computer‐generated list of random numbers.* |
|  | Allocation concealment (selection bias) | Low risk | Intervention was web-based. No contact happened between participant and researcher. |
|  | Blinding of participants and personnel (performance bias) | Low risk | Intervention was web-based delivery. |
|  | Blinding of outcome assessment (detection bias) | Low risk | Outcome assessment was completed by participants themselves online. |
|  | Incomplete outcome data (attrition bias) | Low risk | Judgment was based on the original text: ...*There was no significant difference in % saturated fat intake between participants who only completed the week 1 assessment and those who completed the week 5 measures…* |
|  | Selective reporting (reporting bias) | Low risk | Original data and protocol were provided. |
| Soureti et al. 2011b | Random sequence generation (selection bias) | Low risk | Computer‐generated list of random numbers was used. |
|  | Allocation concealment (selection bias) | Low risk | Intervention was web-based. No contact happened between participant and researcher. |
|  | Blinding of participants and personnel (performance bias) | Low risk | Intervention was web-based delivery. |
|  | Blinding of outcome assessment (detection bias) | Low risk | Outcome assessment was completed by participants themselves online. |
|  | Incomplete outcome data (attrition bias) | Low risk | Intention-to-treat analysis was conducted. |
|  | Selective reporting (reporting bias) | Low risk | Original data and protocol were provided. |
| Stevens et al. 2001 | Random sequence generation (selection bias) | Low risk | Judgment was based on the original text: ...*No substantial imbalances in the distribution of key variables measured at baseline.* |
|  | Allocation concealment (selection bias) | Unclear risk | No relative description. |
|  | Blinding of participants and personnel (performance bias) | Unclear risk | No relative description. |
|  | Blinding of outcome assessment (detection bias) | Low risk | Judgment was based on original text: ...*Clinic staff who were blinded to study group assignment made these assessments.* |
|  | Incomplete outcome data (attrition bias) | Low risk | Judgment was based on original text: ...*Rates of data collection at 36 months were high…* |
|  | Selective reporting (reporting bias) | High risk | No original data. |
| Ströbl et al. 2013 | Random sequence generation (selection bias) | High risk | Judgment was based on original text: ...*Baseline imbalance was observed regarding physical activity, one of the primary outcomes.* |
|  | Allocation concealment (selection bias) | Low risk | Judgment was based on the original text: ...*After having recruited a participant, clinic staff requested the randomization result from the scientific staff by phone (telephone randomization) thus guaranteeing concealment of randomization up to recruitment.* |
|  | Blinding of participants and personnel (performance bias) | Unclear risk | No relative description. |
|  | Blinding of outcome assessment (detection bias) | Unclear risk | No relative description. |
|  | Incomplete outcome data (attrition bias) | Low risk | Intention-to-treat analysis was conducted. |
|  | Selective reporting (reporting bias) | Low risk | Original data and protocol were provided. |
| Svetkey et al. 2008 | Random sequence generation (selection bias) | Low risk | Judgment was based on the original text: ...*Randomization assignments were stratified by clinic, race and amount of weight loss during phase 1 and were allocated in blocks of varying sizes to provide a balance in treatment assignments over time* |
|  | Allocation concealment (selection bias) | Low risk | Judgment was based on the original text: ...*The actual allocation assignments were generated using a password-restricted, Web-based application developed by the coordinating center and were accessible only to authorized unblinded personnel.* |
|  | Blinding of participants and personnel (performance bias) | Low risk | Intervention was web-based delivery. |
|  | Blinding of outcome assessment (detection bias) | Low risk | Judgment was based on original text: ...*Measurements were obtained by trained, certified staff members who were masked to treatment assignment.* |
|  | Incomplete outcome data (attrition bias) | Low risk | Judgment was based on the original text: ...*There were no notable differences in participant characteristics at entry into the study between those who completed the final data-collection visit and the 68 individuals who did not …* |
|  | Selective reporting (reporting bias) | High risk | No original data. |
| Thoolen et al. 2009 | Random sequence generation (selection bias) | Low risk | Judgment was based on the original text: ...*Randomization occurred within each general practice, based on a computer-generated assignment.* |
|  | Allocation concealment (selection bias) | Unclear risk | No relative description. |
|  | Blinding of participants and personnel (performance bias) | High risk | Judgment was based on original text: ...*Not blinded for either the patients or investigators.* |
|  | Blinding of outcome assessment (detection bias) | Unclear risk | No relative description. |
|  | Incomplete outcome data (attrition bias) | Low risk | Judgment was based on the original text: ...*Patients did not differ significantly from participants on any other patient characteristic.* Intention-to-treat analysis was conducted. |
|  | Selective reporting (reporting bias) | Low risk | Original data and protocol were provided. |
| van Genugten et al. 2012 | Random sequence generation (selection bias) | Low risk | Judgment was based on the original text: ...*After baseline assessment, participants were allocated to one of the two study groups (1:1) by means of sex-stratified computer block randomization…* |
|  | Allocation concealment (selection bias) | Low risk | Intervention was web-based. No contact happened between participant and researcher. |
|  | Blinding of participants and personnel (performance bias) | Low risk | Intervention was web-based delivery. |
|  | Blinding of outcome assessment (detection bias) | Unclear risk | No relative description. |
|  | Incomplete outcome data (attrition bias) | High risk | Judgment was based on the original text: ...*Younger people were more likely to drop out between the two moments of anthropometric measures.* |
|  | Selective reporting (reporting bias) | Unclear risk | Original data was provided without protocol. |
| Vinkers et al. 2014 | Random sequence generation (selection bias) | Low risk | Judgment was based on the original text: ...*Participants were allocated to conditions using the randomization function in Excel, and assigned by the first author.* |
|  | Allocation concealment (selection bias) | Unclear risk | No relative description. |
|  | Blinding of participants and personnel (performance bias) | Low risk | Judgment was based on original text: ...*Each trainer led only one type of booster sessions (i.e., they were blinded to the existence of different versions of booster sessions) …* |
|  | Blinding of outcome assessment (detection bias) | Unclear risk | No relative description. |
|  | Incomplete outcome data (attrition bias) | Low risk | Judgment was based on the original text: ...*Drop outs and completers, regardless of timing of drop out, did not differ on any baseline measures…* Intention-to-treat analysis was conducted. |
|  | Selective reporting (reporting bias) | Low risk | Original data and protocol were provided. |
| Wilczynska et al. 2019 | Random sequence generation (selection bias) | Low risk | Judgment was based on the original text: ...*Random allocation to the wait-list control group or the intervention group was performed using a computer-based random number producing algorithm by a researcher not involved in the present study.* |
|  | Allocation concealment (selection bias) | Low risk | Judgment was based on the original text: Random *allocation was performed using a computer-based random number producing algorithm by a researcher not involved in the present study…Participants had an equal chance of allocation to each group.* |
|  | Blinding of participants and personnel (performance bias) | High risk | Single-blind. |
|  | Blinding of outcome assessment (detection bias) | Unclear risk | No relative description. |
|  | Incomplete outcome data (attrition bias) | Low risk | Intention-to-treat analysis was conducted. |
|  | Selective reporting (reporting bias) | Low risk | Original data and protocol were provided. |
| Wooldridge et al. 2019 | Random sequence generation (selection bias) | Low risk | Judgment was based on the original text: ...*research assistants randomized couples using a computer-generated list of random numbers to the collaborative IIs condition, individual IIs condition, or the control condition…* |
|  | Allocation concealment (selection bias) | Low risk | Judgment was based on the original text: ...*The intervention and assessments were completed through mailings and online surveys, and research staff did not interact with participants directly after initial eligibility screening.* |
|  | Blinding of participants and personnel (performance bias) | High risk | Single-blind. |
|  | Blinding of outcome assessment (detection bias) | Low risk | Judgment was based on original text: ...*The intervention and assessments were completed through mailings and online surveys, and research staff did not interact with participants directly after initial eligibility screening.* |
|  | Incomplete outcome data (attrition bias) | Low risk | Judgment was based on original text: *Retention rates did not differ by study condition…* |
|  | Selective reporting (reporting bias) | Unclear risk | Original data was provided without protocol. |
| Zakrisson et al. 2019 | Random sequence generation (selection bias) | Low risk | Judgment was based on the original text: ...*the patients were randomized in separate diagnosis groups (block randomization) to an intervention or a control group at each PHC center.* |
|  | Allocation concealment (selection bias) | Low risk | Judgment was based on the original text: ...*Coded randomizations were performed by the blinded research leader and the code was passed to the district nurse.* |
|  | Blinding of participants and personnel (performance bias) | Low risk | Judgment was based on original text: ...*rapid double-blind peer review with constructive feedback.* |
|  | Blinding of outcome assessment (detection bias) | Low risk | Judgment was based on original text: ...*An independent nurse or a physiotherapist performed all the assessments at the PHC centers that were blinded for the patients’ randomization* |
|  | Incomplete outcome data (attrition bias) | Unclear risk | No analysis was conducted for dropouts. |
|  | Selective reporting (reporting bias) | High risk | No original data. |
| Zandstra et al. 2010 | Random sequence generation (selection bias) | Low risk | Judgment was based on the original text: ...*The initial difference in weight between participants in the implementation intention group and control group was not significant…* |
|  | Allocation concealment (selection bias) | Unclear risk | No relative description. |
|  | Blinding of participants and personnel (performance bias) | Unclear risk | No relative description. |
|  | Blinding of outcome assessment (detection bias) | Unclear risk | No relative description. |
|  | Incomplete outcome data (attrition bias) | Low risk | Judgment was based on the original text: ...*During the study, six subjects dropped out for personal reasons: their data were excluded from statistical analysis.* |
|  | Selective reporting (reporting bias) | High risk | No original data. |
| Armitage et al. 2014 | Random sequence generation (selection bias) | Low risk | Judgment was based on the original text: *...The interventions were placed at the end of identical-looking questionnaires, which were sorted into random order using coin tosses by the researcher prior to data collection.* |
|  | Allocation concealment (selection bias) | Low risk | Judgment was based on the original text: *...Once the pre-randomization questionnaire was completed, the participant returned it to the researcher in a sealed envelope.* |
|  | Blinding of participants and personnel (performance bias) | Unclear risk | No relative description. |
|  | Blinding of outcome assessment (detection bias) | Low risk | Judgment was based on the original text: ...*At follow-up, weight (kg) was again measured independently of the research team, meaning that assessment was carried out blind to condition.* |
|  | Incomplete outcome data (attrition bias) | Low risk | Judgment was based on the original text: ..*.There was no differential drop-out between conditions.* Intention-to-treat analysis was conducted. |
|  | Selective reporting (reporting bias) | Unclear risk | Original data was provided without protocol. |
| Armitage et al. 2017 | Random sequence generation (selection bias) | Low risk | Judgment was based on the original text: ..*.Questionnaires with the intervention/control instructions were sorted into random order using coin tosses by the researcher prior to data collection* |
|  | Allocation concealment (selection bias) | Low risk | Judgment was based on the original text: ..*.Once the baseline questionnaire was completed, the participant returned it to the receptionist in a sealed opaque envelope. Receptionists then distributed these identical-looking questionnaires and participants were left alone to complete the baseline questionnaire in the clinic.* |
|  | Blinding of participants and personnel (performance bias) | Unclear risk | No relative description. |
|  | Blinding of outcome assessment (detection bias) | Low risk | Judgment was based on the original text: ...*The people who weighed participants at baseline and follow-up were blind with respect to condition.* |
|  | Incomplete outcome data (attrition bias) | High risk | Judgment was based on the original text: ...*Significantly more people dropped out of the control group than the intervention group and those who dropped out were taller than those who remained in the study.* |
|  | Selective reporting (reporting bias) | Unclear risk | Original data was provided without protocol. |
| Silva et al. 2017 | Random sequence generation (selection bias) | Low risk | Judgment was based on the original text: *a random allocation list was created, applying a series of random, computer generated numbers that selected 68 participants among 405 eligible individuals.* |
|  | Allocation concealment (selection bias) | Low risk | Judgment was based on the original text: *They were invited by the researcher, via telephone, to participate in the study.* |
|  | Blinding of participants and personnel (performance bias) | Unclear risk | No relative description. |
|  | Blinding of outcome assessment (detection bias) | Low risk | Judgment was based on the original text: *The researchers were blinded by anthropometric measurements and the filling of the instruments.* |
|  | Incomplete outcome data (attrition bias) | Low risk | Judgment was based on the original text: *Over the twelve months of intervention, there were three patients lost to follow-up (CG – two losses due to death and use of drugs/IG – one loss due to death)* |
|  | Selective reporting (reporting bias) | High risk | No original data. |
| Silva et al. 2020 | Random sequence generation (selection bias) | Low risk | Judgment was based on the original text: *…a random allocation list was created, applying a series of random, computer-generated numbers* |
|  | Allocation concealment (selection bias) | Low risk |  |
|  | Blinding of participants and personnel (performance bias) | High risk |  |
|  | Blinding of outcome assessment (detection bias) | Low risk |  |
|  | Incomplete outcome data (attrition bias) | Unclear risk |  |
|  | Selective reporting (reporting bias) | Unclear risk |  |
| Nishita et al. 2013 | Random sequence generation (selection bias) | Low risk | Judgment was based on the original text: *A blocked design was used so that the random assignment of individuals would result in as close to a 2:1 allocation as possible, regardless of the number of individuals that enrolled in the study.* |
|  | Allocation concealment (selection bias) | Low risk | Judgment was based on the original text: ...*individual assignments were placed in sealed envelopes by the data manager.* |
|  | Blinding of participants and personnel (performance bias) | Unclear risk | No relative description. |
|  | Blinding of outcome assessment (detection bias) | Low risk | Outcome assessment was mailed and completed by participants themselves at home. |
|  | Incomplete outcome data (attrition bias) | Unclear risk | No analysis was conducted for dropouts. |
|  | Selective reporting (reporting bias) | Unclear risk | Original data was provided without protocol. |
| Abdolkarimi et al. 2021 | Random sequence generation (selection bias) | Unclear risk | Judgment was based on the original text: *According to their file number and random sampling method, the eligible individuals were entered the study to reach the sample size.* |
|  | Allocation concealment (selection bias) | Unclear risk | No relative description. |
|  | Blinding of participants and personnel (performance bias) | Unclear risk | No relative description. |
|  | Blinding of outcome assessment (detection bias) | Unclear risk | No relative description. |
|  | Incomplete outcome data (attrition bias) | Unclear risk | No analysis was conducted for dropouts. |
|  | Selective reporting (reporting bias) | Low risk | Original data and protocol were provided. |
| Li et al. 2019 | Random sequence generation (selection bias) | Low risk | Judgment was based on the original text:...*随机分成两组* |
|  | Allocation concealment (selection bias) | Unclear risk | No relative description. |
|  | Blinding of participants and personnel (performance bias) | Unclear risk | No relative description. |
|  | Blinding of outcome assessment (detection bias) | Unclear risk | No relative description. |
|  | Incomplete outcome data (attrition bias) | Unclear risk | No analysis was conducted for dropouts. |
|  | Selective reporting (reporting bias) | Unclear risk | Original data was provided without protocol. |
| MacPhail et al. 2014 | Random sequence generation (selection bias) | Unclear risk | Judgment was based on the original text: ...*Participants were then randomly allocated, via a random numbers table,…* |
|  | Allocation concealment (selection bias) | Low risk | Judgment was based on the original text: ...*Treatment group allocation was concealed until after participants had completed the assessment* |
|  | Blinding of participants and personnel (performance bias) | High risk | Judgment was based on original text: ...*Treating doctors and nurses remained blind to the allocation of participants to groups; however, due to the nature of the study, the researcher was not blinded to the treatment type.* |
|  | Blinding of outcome assessment (detection bias) | High risk | Judgment was based on the original text: ...*the (assessment) researcher was not blinded to the treatment type.* |
|  | Incomplete outcome data (attrition bias) | Low risk | Judgment was based on the original text: ..*.There were no differences between intervention completers and those who dropped out on any of the demographic* |
|  | Selective reporting (reporting bias) | High risk | No original data. |
| Hardeman et al. 2009 | Random sequence generation (selection bias) | Low risk | Judgment was based on the original text: ...*participants were randomised. This was carried out centrally by the trial statistician, incorporating a partial minimisation procedure that dynamically adjusted randomisation probabilities to balance key baseline covariates.* |
|  | Allocation concealment (selection bias) | Unclear risk | No relative description. |
|  | Blinding of participants and personnel (performance bias) | Unclear risk | No relative description. |
|  | Blinding of outcome assessment (detection bias) | Unclear risk | No relative description. |
|  | Incomplete outcome data (attrition bias) | Unclear risk | No analysis was conducted for dropouts. |
|  | Selective reporting (reporting bias) | High risk | No original data. |
| Heredia et al. 2019 | Random sequence generation (selection bias) | Low risk | Judgment was based on the original text: ...*A total of 168 patients were randomly assigned to the intervention (n=84) or control group (n=84) using minimization randomization* |
|  | Allocation concealment (selection bias) | Unclear risk | No relative description. |
|  | Blinding of participants and personnel (performance bias) | Unclear risk | No relative description. |
|  | Blinding of outcome assessment (detection bias) | Low risk | Outcome assessment was completed by participants themselves online. |
|  | Incomplete outcome data (attrition bias) | Low risk | Judgment was based on the original text: ..*.We explored drop-outs and missing data, which are common problems with longitudinal data.* |
|  | Selective reporting (reporting bias) | High risk | No original data. |
| Heideman et al. 2015 | Random sequence generation (selection bias) | Unclear risk | Judgment was based on the original text: ...*After signing informed consent, participants were randomly assigned to the intervention or control group* |
|  | Allocation concealment (selection bias) | Low risk | Judgment was based on the original text: ...*The concealed allocation sequence was generated by an independent researcher with serially numbered sealed envelopes.* |
|  | Blinding of participants and personnel (performance bias) | High risk | Judgment was based on original text: ...*Participants and trainers could not be blinded to treatment group because of the nature of the intervention.* |
|  | Blinding of outcome assessment (detection bias) | Unclear risk | No relative description. |
|  | Incomplete outcome data (attrition bias) | Low risk | Judgment was based on the original text: ..*.Eight participants dropped out after randomization, before the first appointment and 21 individuals were excluded after baseline measurement because they did not meet inclusion criteria.* |
|  | Selective reporting (reporting bias) | High risk | No original data. |
| Engel et al. 2006 | Random sequence generation (selection bias) | Low risk | Judgment was based on the original text: ...*participants were randomized to either the pedometer (intervention) or coaching-only (control) group using random number tables* |
|  | Allocation concealment (selection bias) | High risk | Judgment was based on the original text: ...*once randomization was completed, the principal investigator was aware to which treatment groups participants had been allocated.* |
|  | Blinding of participants and personnel (performance bias) | High risk | Judgment was based on original text: ...*Participants were told to which group they had been allocated ....the principal investigator was aware to which treatment groups participants had been allocated.* |
|  | Blinding of outcome assessment (detection bias) | High risk | Judgment was based on the original text: ...*the principal investigator was aware to which treatment groups participants had been allocated.* |
|  | Incomplete outcome data (attrition bias) | Low risk | Judgment was based on the original text: ..*.This comprised a 12% dropout rate. Reasons for withdrawal included health problems (unrelated to increased walking) and work commitments.* |
|  | Selective reporting (reporting bias) | High risk | No original data. |
| Liu et al. 2015 | Random sequence generation (selection bias) | Unclear risk | Judgment was based on the original text: ...*将患者随机分为试验组50 例和对照组50例* |
|  | Allocation concealment (selection bias) | Unclear risk | No relative description. |
|  | Blinding of participants and personnel (performance bias) | Unclear risk | No relative description. |
|  | Blinding of outcome assessment (detection bias) | Unclear risk | No relative description. |
|  | Incomplete outcome data (attrition bias) | Unclear risk | No analysis was conducted for dropouts. |
|  | Selective reporting (reporting bias) | Unclear risk | Original data was provided without protocol. |
| Gao et al. 2006 | Random sequence generation (selection bias) | Low risk | Judgment was based on the original text: ...*将 80例初发2型糖尿病患者按随机数字表法分为观察组和对照组各 40 例* |
|  | Allocation concealment (selection bias) | Unclear risk | No relative description. |
|  | Blinding of participants and personnel (performance bias) | Unclear risk | No relative description. |
|  | Blinding of outcome assessment (detection bias) | Unclear risk | No relative description. |
|  | Incomplete outcome data (attrition bias) | Low risk | Judgment was based on the original text: *对照组 3 例失访， 其中 2 例定期电话联系 3 次均未 接通失联、1 例因骨折不愿意继续参加随访，观察组 2 例因为路途较远不愿意再接受随访* |
|  | Selective reporting (reporting bias) | Unclear risk | Original data was provided without protocol. |
| Mayer et al. 2019 | Random sequence generation (selection bias) | Low risk | Judgment was based on the original text: ...*allocated participants to intervention or control (delayed intervention) groups using cluster, blinded randomization for each enrollment site to account for site differences.* |
|  | Allocation concealment (selection bias) | Low risk | Judgment was based on the original text: ...*allocated participants to intervention or control (delayed intervention) groups using cluster, blinded randomization for each enrollment site to account for site differences.* |
|  | Blinding of participants and personnel (performance bias) | Low risk | Judgment was based on original text: ...*blinded randomization* |
|  | Blinding of outcome assessment (detection bias) | Low risk | Judgment was based on the original text: ...*assessments conducted by research assistants blinded to their group assignment.* |
|  | Incomplete outcome data (attrition bias) | Low risk | Intention-to-treat analysis was conducted. |
|  | Selective reporting (reporting bias) | Unclear risk | Original data was provided without protocol. |
| Wurst et al. 2019 | Random sequence generation (selection bias) | Low risk | Judgment was based on the original text: ...*A randomization list was created by a researcher (SK) before trial begin using Excel 2007* |
|  | Allocation concealment (selection bias) | Low risk | Judgment was based on the original text: ...*the clinical staff not involved in the intervention or study accessed the randomization list and conducted the allocation for the included participants.* |
|  | Blinding of participants and personnel (performance bias) | Unclear risk | No relative description. |
|  | Blinding of outcome assessment (detection bias) | Low risk | Outcome assessment was mailed and completed by participants themselves at home. |
|  | Incomplete outcome data (attrition bias) | Low risk | Intention-to-treat analysis was conducted. |
|  | Selective reporting (reporting bias) | Low risk | Original data and protocol were provided. |
| Su et al. 2021 | Random sequence generation (selection bias) | Low risk | Judgment was based on the original text: ...*Random numbers and group allocation codes were sealed in opaque envelopes* |
|  | Allocation concealment (selection bias) | Low risk | Judgment was based on the original text: ...*Random numbers and group allocation codes were sealed in opaque envelopes* |
|  | Blinding of participants and personnel (performance bias) | High risk | Single-blind. |
|  | Blinding of outcome assessment (detection bias) | Low risk | Judgment was based on the original text: ...*Post-test data were collected at 6 and 12 weeks post-intervention via face-to-face interviews conducted by research assistants who had no information of group assignment.* |
|  | Incomplete outcome data (attrition bias) | Low risk | Reasons for drop-outs were shown. |
|  | Selective reporting (reporting bias) | Low risk | Original data and protocol were provided. |
| Washington et al. 2021 | Random sequence generation (selection bias) | Low risk | Judgment was based on the original text: ...*We used variable-block size randomization algorithm* |
|  | Allocation concealment (selection bias) | Low risk | Judgment was based on the original text: ...We used variable-block size randomization algorithm to eliminate selection bias and to ensure intermittent |
|  | Blinding of participants and personnel (performance bias) | Unclear risk | No relative description. |
|  | Blinding of outcome assessment (detection bias) | Unclear risk | No relative description. |
|  | Incomplete outcome data (attrition bias) | Low risk | Reasons for drop-outs were shown. |
|  | Selective reporting (reporting bias) | Low risk | Original data and protocol were provided. |
| Peacock et al. 2020 | Random sequence generation (selection bias) | Low risk | Judgment was based on the original text: ...*Individual patients were the unit of randomisation,...* |
|  | Allocation concealment (selection bias) | Low risk | Judgment was based on the original text: ...*Participants were allocated remotely by the trial statistician via concealed minimisation* |
|  | Blinding of participants and personnel (performance bias) | High risk | Single-blind. |
|  | Blinding of outcome assessment (detection bias) | Low risk | Judgment was based on the original text: ...*Researchers assessing the primary outcomes were blinded to the allocation of participants.* |
|  | Incomplete outcome data (attrition bias) | Low risk | Intention-to-treat analysis was conducted. |
|  | Selective reporting (reporting bias) | Low risk | Original data and protocol were provided. |
| Kuijer et al. 2007 | Random sequence generation (selection bias) | Low risk | Judgment was based on the original text: ...*Patients were randomly allocated to the experimental group (intervention and standard care) and control group* |
|  | Allocation concealment (selection bias) | Unclear risk | No relative description. |
|  | Blinding of participants and personnel (performance bias) | Unclear risk | No relative description. |
|  | Blinding of outcome assessment (detection bias) | Low risk | Judgment was based on the original text: ...*Two weeks (post-test; T2) and 6 months (follow-up; T3) after the intervention, they received questionnaires to complete at home.* |
|  | Incomplete outcome data (attrition bias) | Low risk | Intention-to-treat analysis was conducted. |
|  | Selective reporting (reporting bias) | Unclear risk | Original data was provided without protocol. |
| Eakin et al. 2009 | Random sequence generation (selection bias) | Unclear risk | Judgment was based on the original text: ...*Ten clinics were assigned by simple random allocation using a computer generated random number table to either the telephone counseling intervention or to usual care.* |
|  | Allocation concealment (selection bias) | Unclear risk | No relative description. |
|  | Blinding of participants and personnel (performance bias) | Unclear risk | No relative description. |
|  | Blinding of outcome assessment (detection bias) | Low risk | Judgment was based on the original text: *All study outcomes were obtained using computer-assisted telephone interviews at baseline, 4, and 12 months, by interviewers who were blind to study condition* |
|  | Incomplete outcome data (attrition bias) | Low risk | Intention-to-treat analysis was conducted. |
|  | Selective reporting (reporting bias) | High risk | No original data. |
| Jiang et al. 2021 | Random sequence generation (selection bias) | Low risk | Judgment was based on the original text: ...*Participants were randomly assigned to either intervention (group n = 17) or control group using SAS software to generate a random sequence…* |
|  | Allocation concealment (selection bias) | Low risk | Judgment was based on the original text: ...*The result of the allocation sequence was printed and placed in an opaque envelope and sealed.* |
|  | Blinding of participants and personnel (performance bias) | Low risk | Judgment was based on original text: ...*Patients in the intervention group and the control group did not know each other. Different general practitioners were assigned to be responsible for the service provided for the two groups, and the time for the two groups of patients to receive services was arranged at different days of the week* |
|  | Blinding of outcome assessment (detection bias) | Low risk | Judgment was based on the original text: ...*The allocation sequence was concealed from the researchers responsible for recruitment and result evaluation.* |
|  | Incomplete outcome data (attrition bias) | Low risk | Judgment was based on the original text: ..*.There was no significant difference between the missing subjects and the baseline subjects in gender, age, and the history of the diabetes* |
|  | Selective reporting (reporting bias) | Low risk | Original data and protocol were provided. |
| Swoboda et al. 2016 | Random sequence generation (selection bias) | Low risk | Judgment was based on the original text: *Randomization envelopes were prepared by a statistician and retained in opaque envelopes until revealed to each participant by the study coordinator.* |
|  | Allocation concealment (selection bias) | Low risk | Judgment was based on the original text: ...*Randomization envelopes were prepared by a statistician and retained in opaque envelopes until revealed to each participant by the study coordinator.* |
|  | Blinding of participants and personnel (performance bias) | High risk | Single-blind. |
|  | Blinding of outcome assessment (detection bias) | Unclear risk | No relative description. |
|  | Incomplete outcome data (attrition bias) | Low risk | Reasons for drop-outs were shown. |
|  | Selective reporting (reporting bias) | Low risk | Original data and protocol were provided. |
