# Supplementary material for: Making Specific Plan Improves Physical Activity and Healthy Eating for Community-Dwelling Patients With Chronic Conditions: A Systematic Review and Meta-Analysis
Source: Front Public Health. 2022 May 19;10:721223. doi: 10.3389/fpubh.2022.721223 (PMC9160833; doi:10.3389/fpubh.2022.721223)
Supplement: Supplementary file 4 [file Table_4.DOCX]

Supplementary Material

# Supplemental Table 4. Extracting outcome data for meta-analyses

| **Outcome** | **Study** | **Experimental** | | | **Control** | | | **MD/SMD 95%CI** |
| --- | --- | --- | --- | --- | --- | --- | --- | --- |
|  |  | **Mean** | **SD** | **Total** | **Mean** | **SD** | **Total** |  |
| Physical activity | Bélanger-Gravel 2013 | 8695.0 | 3447.0 | 33 | 7811.0 | 3191.0 | 36 | 0.26 [-0.21, 0.74] |
|  | Cheung 2017 | 112.24 | 101.42 | 364 | 114.99 | 116.51 | 342 | -0.03 [-0.17, 0.12] |
|  | Eakin 2009 | 195.7 | 240.5 | 228 | 178.6 | 212.2 | 206 | 0.08 [-0.11, 0.26] |
|  | Hardeman 2009 | 1.94 | 0.65 | 224 | 2.0 | 0.57 | 121 | -0.10 [-0.32, 0.13] |
|  | Helena 2014 | 8582.0 | 3748.0 | 33 | 7850.0 | 3764.0 | 30 | 0.19 [-0.30, 0.69] |
|  | Janssen 2014 | 9630.0 | 3598.0 | 89 | 7337.0 | 3767.0 | 86 | 0.62 [0.32, 0.92] |
|  | Kuijer 2007 (1) | 4.25 | 2.06 | 25 | 3.39 | 2.52 | 21 | 0.37 [-0.21, 0.96] |
|  | Kuijer 2007 (2) | 3.87 | 2.22 | 20 | 3.13 | 1.92 | 12 | 0.34 [-0.38, 1.06] |
|  | Luszczynska 2006 | 4.75 | 1.83 | 59 | 4.09 | 1.69 | 55 | 0.37 [0.00, 0.74] |
|  | Miura 2004 | 193.0 | 65.0 | 18 | 172.0 | 106.06 | 19 | 0.23 [-0.41, 0.88] |
|  | Rodrigues 2013 | 3.2 | 0.8 | 69 | 2.8 | 0.9 | 67 | 0.47 [0.13, 0.81] |
|  | Sniehotta 2011 | 38.44 | 21.89 | 20 | 41.0 | 31.51 | 11 | -0.10 [-0.83, 0.64] |
|  | Ströbl 2013 | 9.24 | 6.1 | 228 | 8.5 | 0.4 | 239 | 0.17 [-0.01, 0.35] |
|  | Su 2021 | 9502.29 | 1453.7 | 73 | 7298.04 | 3727.92 | 73 | 0.77 [0.44, 1.11] |
|  | Thoolen 2009 | 4.6 | 1.8 | 78 | 3.3 | 1.9 | 102 | 0.70 [0.39, 1.00] |
|  | van Genugten 2012 | 63.3 | 53.6 | 177 | 78.7 | 60.7 | 184 | -0.27 [-0.48, -0.06] |
|  | Washington 2021 | 349.9 | 930.47 | 58 | 197.9 | 310.72 | 62 | 0.22 [-0.14, 0.58] |
|  | Wilczynska 2019 | 33.1 | 7.18 | 42 | 33.4 | 6.33 | 42 | -0.04 [-0.47, 0.38] |
|  | Wooldridge 2019 | 1783.93 | 2003.94 | 28 | 659.9 | 623.78 | 6 | 0.59 [-0.30, 1.49] |
|  | Wurst 2019 | 206.0 | 211.0 | 71 | 128.0 | 161.0 | 69 | 0.41 [0.08, 0.75] |
|  | Overall |  |  | 1937 |  |  | 1783 | 0.24 [0.10, 0.39] |
| Diet Behavior | Cheung 2017 | 1002.46 | 466.5 | 367 | 1189.77 | 548.16 | 350 | -0.37 [-0.52, -0.22] |
|  | de Freitas Agondi 2014 | 5.5 | 3.2 | 49 | 7.9 | 6.8 | 49 | -0.45 [-0.85, -0.05] |
|  | Eakin 2009 | 34.87 | 5.29 | 228 | 36.45 | 5.81 | 206 | -0.28 [-0.47, -0.10] |
|  | Gao 2019 | -57.53 | 7.43 | 40 | -51.9 | 5.53 | 40 | -0.85 [-1.31, -0.39] |
|  | Jackson 2005 | -4.22 | 2.55 | 30 | -4.08 | 2.01 | 32 | -0.06 [-0.56, 0.44] |
|  | Janssen 2014 | 16.4 | 5.8 | 89 | 16.9 | 5.9 | 87 | -0.09 [-0.38, 0.21] |
|  | Kuijer 2007 | -4.78 | 1.54 | 20 | -5.2 | 1.86 | 12 | 0.25 [-0.47, 0.96] |
|  | Luszczynska, Scholz 2007 | 19.71 | 4.63 | 57 | 22.47 | 5.22 | 57 | -0.56 [-0.93, -0.18] |
|  | Mayer 2019 | 6.16 | 0.84 | 210 | 6.17 | 0.88 | 192 | -0.01 [-0.21, 0.18] |
|  | Miura 2004 (1) | 1807.0 | 202.76 | 20 | 1908.0 | 150.93 | 10 | -0.52 [-1.30, 0.25] |
|  | Miura 2004 (2) | 1782.0 | 247.13 | 17 | 1908.0 | 150.93 | 9 | -0.56 [-1.38, 0.27] |
|  | Obara-Golebiowska 2015 | -6.6 | 0.48 | 50 | -6.28 | 0.57 | 50 | -0.60 [-1.00, -0.20] |
|  | Sniehotta 2011 | 73.59 | 106.62 | 16 | 117.78 | 39.87 | 9 | -0.48 [-1.31, 0.35] |
|  | Soureti 2011a (3) | -4.98 | 1.03 | 194 | -4.86 | 0.98 | 97 | -0.12 [-0.36, 0.13] |
|  | Soureti 2011a (4) | -5.09 | 1.06 | 195 | -4.86 | 0.98 | 98 | -0.22 [-0.47, 0.02] |
|  | Soureti 2011b (5) | 3.6 | 5.11 | 254 | 3.9 | 3.8 | 131 | -0.06 [-0.27, 0.15] |
|  | Soureti 2011b (6) | 3.5 | 4.06 | 251 | 3.9 | 3.8 | 131 | -0.10 [-0.31, 0.11] |
|  | Swoboda 2016 | 1876.65 | 1860.0 | 34 | 2037.07 | 667.16 | 15 | -0.10 [-0.71, 0.51] |
|  | Thoolen 2009 | -5.5 | 1.0 | 78 | -4.9 | 1.1 | 102 | -0.56 [-0.87, -0.26] |
|  | van Genugten 2012 | 15.4 | 6.0 | 177 | 15.9 | 6.4 | 184 | -0.08 [-0.29, 0.13] |
|  | Vinkers 2014 | 39.17 | 3.76 | 83 | 39.95 | 3.72 | 60 | -0.21 [-0.54, 0.13] |
|  | Overall |  |  | 2459 |  |  | 1921 | -0.25 [-0.34, -0.15] |
| Weight | Armitage 2014 | 79.42 | 8.46 | 36 | 78.47 | 8.46 | 36 | 0.95 [-2.96, 4.86] |
|  | Armitage 2017 | 81.34 | 15.28 | 119 | 88.06 | 17.84 | 97 | -6.72 [-11.21, -2.23] |
|  | Engel 2006 | 89.8 | 25.1 | 22 | 83.4 | 18.3 | 28 | 6.40 [-6.09, 18.89] |
|  | Heideman 2015 | 85.46 | 18.08 | 42 | 88.58 | 18.44 | 44 | -3.12 [-10.84, 4.60] |
|  | Helena 2014 | 104.5 | 20.0 | 36 | 104.7 | 18.5 | 37 | -0.20 [-9.04, 8.64] |
|  | Jiang 2021 | 68.1 | 10.96 | 251 | 69.65 | 10.28 | 249 | -1.55 [-3.41, 0.31] |
|  | Luszczynska, Sobczyk 2007 | 84.48 | 19.48 | 27 | 87.33 | 21.15 | 28 | -2.85 [-13.59, 7.89] |
|  | Mayer 2019 | 91.4 | 22.47 | 210 | 90.0 | 21.26 | 192 | 1.40 [-2.88, 5.68] |
|  | Sniehotta 2011 | 102.19 | 26.32 | 32 | 92.63 | 19.81 | 21 | 9.56 [-2.89, 22.01] |
|  | Stevens 2001 | 89.03 | 12.22 | 565 | 93.45 | 14.47 | 561 | -4.42 [-5.98, -2.86] |
|  | Svetkey 2008 (1) | 93.8 | 15.7 | 347 | 92.9 | 15.6 | 170 | 0.90 [-1.97, 3.77] |
|  | Svetkey 2008 (2) | 92.7 | 17.2 | 341 | 92.9 | 15.6 | 171 | -0.20 [-3.17, 2.77] |
|  | Overall |  |  | 2028 |  |  | 1634 | -1.13 [-3.02, 0.76] |
| Body Mass Index | Broekhuizen 2012 | 25.8 | 4.4 | 167 | 27.1 | 5.2 | 147 | -1.30 [-2.37, -0.23] |
|  | Bélanger-Gravel 2013 | 33.2 | 3.8 | 36 | 32.5 | 2.5 | 37 | 0.70 [-0.78, 2.18] |
|  | Cheung 2017 | 28.35 | 4.89 | 672 | 28.72 | 5.09 | 393 | -0.37 [-0.99, 0.25] |
|  | Engel 2006 | 32.0 | 7.78 | 22 | 30.5 | 6.1 | 28 | 1.50 [-2.46, 5.46] |
|  | Heideman 2015 | 29.69 | 5.2 | 42 | 31.21 | 5.72 | 44 | -1.52 [-3.83, 0.79] |
|  | Helena 2014 | 34.6 | 5.7 | 36 | 33.5 | 4.3 | 37 | 1.10 [-1.22, 3.42] |
|  | Janssen 2014 | 28.1 | 3.6 | 89 | 28.5 | 4.3 | 86 | -0.40 [-1.58, 0.78] |
|  | Luszczynska, Sobczyk 2007 | 31.07 | 6.25 | 27 | 32.88 | 6.02 | 28 | -1.81 [-5.05, 1.43] |
|  | Mayer 2019 | 32.29 | 6.58 | 210 | 32.34 | 5.63 | 192 | -0.05 [-1.24, 1.14] |
|  | Nishita 2013 | 32.37 | 2.61 | 128 | 33.06 | 2.36 | 62 | -0.69 [-1.43, 0.05] |
|  | Ströbl 2013 | 35.19 | 4.0 | 177 | 35.26 | 3.98 | 164 | -0.07 [-0.92, 0.78] |
|  | Su 2021 | 24.67 | 3.62 | 73 | 24.51 | 2.51 | 73 | 0.16 [-0.85, 1.17] |
|  | Thoolen 2009 | 29.6 | 5.1 | 78 | 30.3 | 5.1 | 102 | -0.70 [-2.20, 0.80] |
|  | van Genugten 2012 | 28.09 | 2.36 | 151 | 27.61 | 2.03 | 161 | 0.48 [-0.01, 0.97] |
|  | Washington 2021 | 34.0 | 8.29 | 58 | 36.3 | 8.48 | 62 | -2.30 [-5.30, 0.70] |
|  | Overall |  |  | 1966 |  |  | 1616 | -0.23 [-0.61, 0.14] |

MD, mean difference; SMD, standard mean difference; CI, confidence interval.
