# Supplementary material for: Making Specific Plan Improves Physical Activity and Healthy Eating for Community-Dwelling Patients With Chronic Conditions: A Systematic Review and Meta-Analysis
Source: Front Public Health. 2022 May 19;10:721223. doi: 10.3389/fpubh.2022.721223 (PMC9160833; doi:10.3389/fpubh.2022.721223)
Supplement: Supplementary file 5 [file Table_5.DOCX]

Supplementary Material

# Supplemental Table 5. Sensitivity analysis results of sequential algorithm

|  | Study Removed | Estimates after removing the study, 95%CI | I^2^ (%) |
| --- | --- | --- | --- |
|  | Bélanger-Gravel 2013 | 0.24 [0.10, 0.39] | 75 |
|  | Cheung 2017 | 0.27 [0.11, 0.42] | 72 |
|  | Eakin 2009 | 0.26 [0.10, 0.41] | 75 |
|  | Hardeman 2009 | 0.27 [0.12, 0.42] | 73 |
|  | Helena 2014 | 0.25 [0.10, 0.39] | 75 |
|  | Janssen 2014 | 0.22 [0.08, 0.36] | 71 |
|  | Kuijer 2007 (1) | 0.24 [0.09, 0.38] | 75 |
|  | Kuijer 2007 (2) | 0.24 [0.10, 0.39] | 75 |
|  | Luszczynska 2006 | 0.24 [0.09, 0.38] | 74 |
|  | Miura 2004 | 0.24 [0.10, 0.39] | 75 |
|  | Rodrigues 2013 | 0.23 [0.09, 0.38] | 74 |
|  | Sniehotta 2011 | 0.25 [0.11, 0.40] | 75 |
|  | Ströbl 2013 | 0.25 [0.10, 0.41] | 75 |
|  | Su 2021 | 0.21 [0.07, 0.34] | 69 |
|  | Thoolen 2009 | 0.21 [0.08, 0.35] | 69 |
|  | van Genugten 2012 | 0.28 [0.14, 0.41] | 67 |
|  | Washington 2021 | 0.25 [0.10, 0.39] | 75 |
|  | Wilczynska 2019 | 0.26 [0.11, 0.40] | 75 |
|  | Wooldridge 2019 | 0.24 [0.09, 0.38] | 75 |
|  | Wurst 2019 | 0.23 [0.09, 0.38] | 74 |
|  | Overall | 0.24 [0.10, 0.39] | 74 |
|  | Cheung 2017 | -0.24 [-0.34, -0.14] | 46 |
|  | de Freitas Agondi 2014 | -0.24 [-0.34, -0.15] | 50 |
|  | Eakin 2009 | -0.25 [-0.35, -0.15] | 51 |
|  | Gao 2019 | -0.23 [-0.31, -0.14] | 40 |
|  | Jackson 2005 | -0.26 [-0.35, -0.16] | 51 |
|  | Janssen 2014 | -0.26 [-0.36, -0.16] | 50 |
|  | Kuijer 2007 | -0.26 [-0.35, -0.16] | 49 |
|  | Luszczynska, Scholz 2007 | -0.23 [-0.33, -0.14] | 47 |
|  | Mayer 2019 | -0.27 [-0.36, -0.17] | 44 |
|  | Miura 2004 (1) | -0.25 [-0.34, -0.15] | 51 |
|  | Miura 2004 (2) | -0.25 [-0.34, -0.15] | 51 |
|  | Obara-Golebiowska 2015 | -0.23 [-0.33, -0.14] | 47 |
|  | Sniehotta 2011 | -0.25 [-0.34, -0.15] | 51 |
|  | Soureti 2011a (1) | -0.26 [-0.36, -0.16] | 50 |
|  | Soureti 2011a (2) | -0.25 [-0.35, -0.15] | 51 |
|  | Soureti 2011b (1) | -0.26 [-0.36, -0.17] | 48 |
|  | Soureti 2011b (2) | -0.26 [-0.36, -0.16] | 49 |
|  | Swoboda 2016 | -0.26 [-0.35, -0.16] | 51 |
|  | Thoolen 2009 | -0.23 [-0.32, -0.14] | 44 |
|  | van Genugten 2012 | -0.26 [-0.36, -0.16] | 48 |
|  | Vinkers 2014 | -0.25 [-0.35, -0.15] | 51 |
|  | Overall | -0.25 [-0.34, -0.15] | 49 |
|  | Armitage 2014 | -1.36 [-3.36, 0.64] | 62 |
|  | Armitage 2017 | -0.63 [-2.52, 1.27] | 59 |
|  | Engel 2006 | -1.29 [-3.18, 0.60] | 63 |
|  | Heideman 2015 | -1.01 [-2.99, 0.97] | 65 |
|  | Helena 2014 | -1.15 [-3.11, 0.82] | 65 |
|  | Jiang 2021 | -0.94 [-3.27, 1.38] | 65 |
|  | Luszczynska, Sobczyk 2007 | -1.06 [-3.02, 0.90] | 65 |
|  | Mayer 2019 | -1.39 [-3.37, 0.58] | 62 |
|  | Sniehotta 2011 | -1.37 [-3.20, 0.46] | 61 |
|  | Stevens 2001 | -0.56 [-2.23, 1.11] | 33 |
|  | Svetkey 2008 (1) | -1.44 [-3.43, 0.55] | 59 |
|  | Svetkey 2008 (2) | -1.22 [-3.32, 0.87] | 63 |
|  | Overall | -1.13 [-3.02, 0.76] | 62 |
|  | Broekhuizen 2012 | -0.12 [-0.47, 0.22] | 23 |
|  | Bélanger-Gravel 2013 | -0.28 [-0.66, 0.10] | 37 |
|  | Cheung 2017 | -0.22 [-0.65, 0.20] | 38 |
|  | Engel 2006 | -0.25 [-0.63, 0.13] | 38 |
|  | Heideman 2015 | -0.20 [-0.58, 0.17] | 36 |
|  | Helena 2014 | -0.27 [-0.64, 0.11] | 37 |
|  | Janssen 2014 | -0.23 [-0.63, 0.17] | 40 |
|  | Luszczynska, Sobczyk 2007 | -0.21 [-0.59, 0.16] | 37 |
|  | Mayer 2019 | -0.25 [-0.66, 0.15] | 40 |
|  | Nishita 2013 | -0.17 [-0.56, 0.22] | 33 |
|  | Ströbl 2013 | -0.26 [-0.68, 0.15] | 40 |
|  | Su 2021 | -0.28 [-0.68, 0.13] | 39 |
|  | Thoolen 2009 | -0.21 [-0.60, 0.18] | 39 |
|  | van Genugten 2012 | -0.38 [-0.69, -0.06] | 2 |
|  | Washington 2021 | -0.20 [-0.56, 0.16] | 34 |
|  | Overall | -0.23 [-0.61, 0.14] | 36 |
