# Supplementary figures and images for: Making Specific Plan Improves Physical Activity and Healthy Eating for Community-Dwelling Patients With Chronic Conditions: A Systematic Review and Meta-Analysis
Source: Front Public Health. 2022 May 19;10:721223. doi: 10.3389/fpubh.2022.721223 (PMC9160833; doi:10.3389/fpubh.2022.721223)

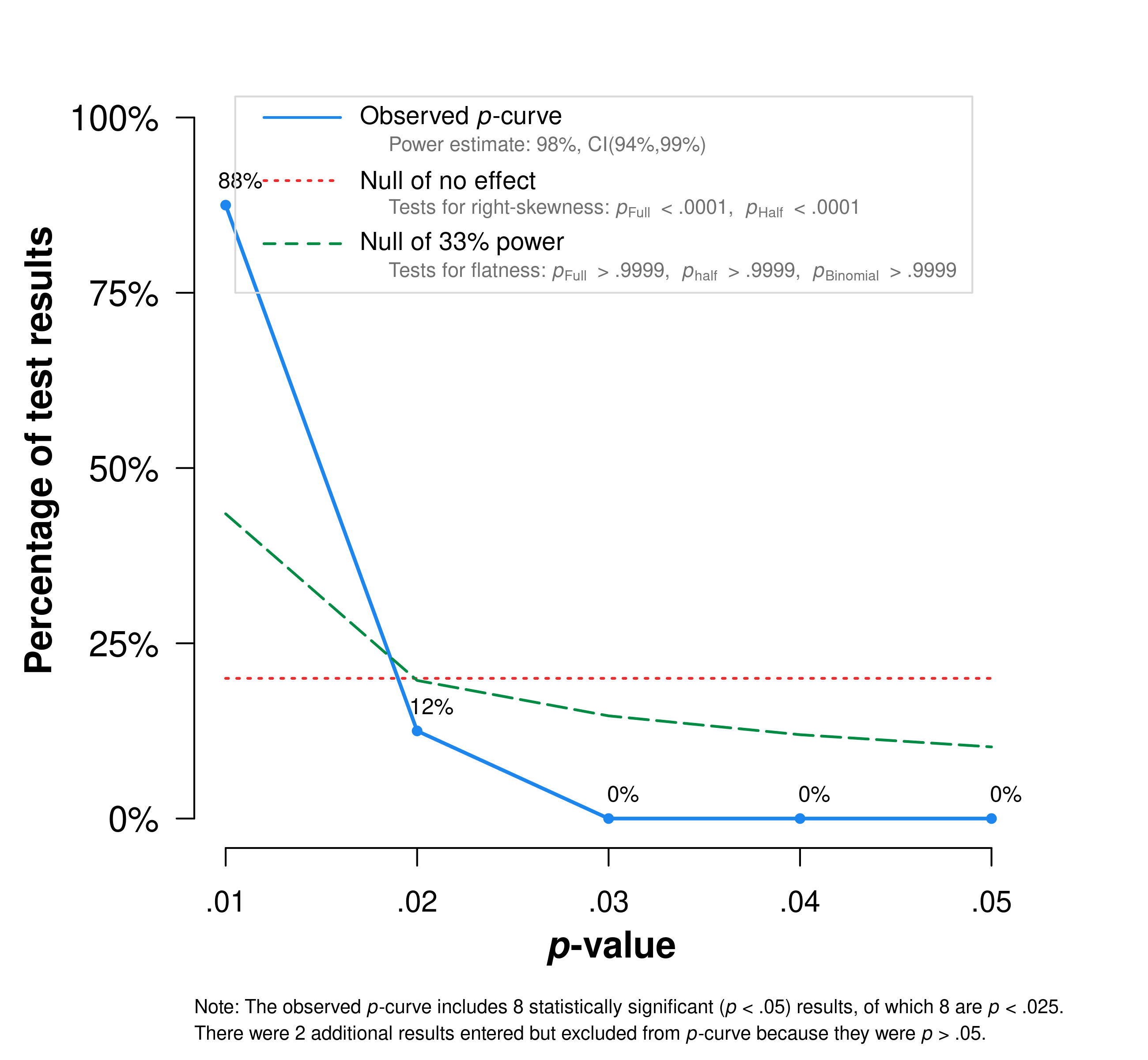

Supplement: Supplementary file 8 [file Image_3.png]
